# Supplementary material for: Two-colour light activated covalent bond formation
Source: Nat Commun. 2022 May 26;13:2943. doi: 10.1038/s41467-022-30002-6 (PMC9135712; doi:10.1038/s41467-022-30002-6)
Supplement: Supplementary file 1 — Supplementary Information [file 41467_2022_30002_MOESM1_ESM.pdf]

Supplementary Information

Two Light Colour Activated Covalent Bond Formation

Sarah L. Walden,‖[a,b] Leona L. Rodrigues,‖[a,b] Jessica Alves,[a,b] James P. Blinco,*[a,b] Vinh X. Truong,*[a,b] Christopher Barner-Kowollik*[a,b,c]

[a]Centre for Materials Science, Queensland University of Technology, 2 George Street, Brisbane, QLD 4000, Australia

[b]School of Physics and Chemistry, Queensland University of Technology, 2 George Street, Brisbane, QLD 4000, Australia

[c]Institute of Nanotechnology, Karlsruhe Institute of Technology (KIT), Hermann-von-Helmholtz-Platz 1, 76344 Eggenstein-Leopoldshafen, Germany

‖ These authors contributed equally

Contents

Supplementary Information 1
1 Experimental details 3
1.1 Bruker 600 MHz NMR 3
1.2 LC-MS 3
1.3 Preparative HPLC 3
1.4 Size Exclusion Chromatography Mass Spectrometry SEC-MS 4
1.5 Size exclusion Chromatography THF-GPC 4
1.6 Shimadzu UV-VIS 5
1.7 LED Characterization 5
1.8 Experiments using LED lamps 5
1.9 In-situ Absorbance 6
1.10 Kinetics and Action Plot with Opolette Laser 7
1.11 Two Color NMR Studies 9
1.12 Rheology Measurements 10
2 Synthesis 11
2.1 Azobenzene A1 11
2.2 Azobenzene A2, A5 13
2.3 Photoactive Ketone 15
2.4 Photoreversible Ketene (K3) 17
2.5 Photoreversible tetra-Ketene (K4) 20
2.6 PEG-(Cl_4-Azobenzene) (A3) 24
2.7 PEG-(Cl_4-Azobenzene)$_3$ crosslinker (A4) 25
2.8 Tri(ethylene glycol) Ketene K5 27
2.9 Photoproducts 33
3 Assessment of photochemical reactivity 43
3.1 Azobenzene 43
3.2 Cl-Azobenzene 44
3.3 Photoactive Ketone 46
3.4 Photoreversible ketene 49
4 Polymer End Group Modification 51

1 Experimental details

1.1 Bruker 600 MHz NMR

^{1}H and ^{13}C-NMR as well as DEPT 135, COSY, HSQC and HMBC-spectra were recorded on a *Bruker* System 600 Ascend LH, equipped with a BBO-Probe (5 mm) with z-gradient (^{1}H: 600.13 MHz, ^{13}C 150.90 MHz). Resonances are reported in parts per million (ppm) relative to tetramethylsilane (TMS). The δ-scale was calibrated to the respective solvent signal of $CHCl_3$ or DCM for ^{1}H spectra and for ^{13}C spectra on the middle signal of the $CDCl_3$ triplet or the DCM singlet. The annotation of the signals is based on HSQC-, COSY- and DEPT-experiments.

1.2 LC-MS

LC-MS measurements were performed on an UltiMate 3000 UHPLC System (Dionex, Sunnyvale, CA, USA) consisting of a pump (LPG 3400SZ), autosampler (WPS 3000TSL) and a temperature controlled column compartment (TCC 3000). Separation was performed on a C18 HPLC column (Phenomenex Luna 5µm, 100 Å, 250 × 2.0 mm) operating at 40 °C. Water (containing 5 mmol L^{-1} ammonium acetate) and acetonitrile were used as eluents. A gradient of acetonitrile:H_2O 5:95 to 100:0 (v/v) in 7 min at a flow rate of 0.40 $mL\cdot min^{-1}$ was applied. The flow was split in a 9:1 ratio, where 90 % of the eluent was directed through a DAD UV-detector (VWD 3400, Dionex) and 10 % was infused into the electrospray source. Spectra were recorded on an LTQ Orbitrap Elite mass spectrometer (Thermo Fisher Scientific, San Jose, CA, USA) equipped with a HESI II probe. The instrument was calibrated in the m/z range 74-1822 using premixed calibration solutions (Thermo Scientific). A constant spray voltage of 3.5 kV, a dimensionless sheath gas and a dimensionless auxiliary gas flow rate of 5 and 2 were applied, respectively. The capillary temperature and was set to 300 °C, the S-lens RF level was set to 68, and the aux gas heater temperature was set to 100 °C.

1.3 Preparative HPLC

Preparative HPLC was performed on an Interchim PF5.250 HPLC system consisting of a SP-in-line filter 20-µm, an UV-VIS detector (200-800 nm) and a Nano-IELSD (45 °C dift tube

temperature) connected *via* a dynamic flow splitter flow splitter. The separations were performed using a direct injection *via* an injection valve and an Interchim Uptisphere Silica HP 5 µm column with 21.2 mm diameter and 250 mm length eq.uipped with a pre-column filled with 5 µm silica.

1.4 Size Exclusion Chromatography Mass Spectrometry SEC-MS

Spectra were recorded on a Q Exactive Plus (Orbitrap) mass spectrometer (Thermo Fisher Scientific, San Jose, CA, USA) equipped with a HESI II probe. The instrument was calibrated in the m/z range 74-1822 using premixed calibration solutions (Thermo Scientific) and for the high mass mode in the m/z range of 600-8000 using ammonium hexafluorophosphate solution. A constant spray voltage of 3.5 kV, a dimensionless sheath gas and a dimensionless auxiliary gas flow rate of 10 and 0 were applied, respectively. The capillary temperature was set to 320 °C, the S-lens RF level was set to 150 and the aux gas heater temperature was set to 125 °C. The Q Exactive was coupled to an UltiMate 3000 UHPLC System (Dionex, Sunnyvale, CA, USA) consisting of a pump (LPG 3400SD), autosampler (WPS 3000TSL), and a temperature-controlled column department (TCC 3000). Separation was performed on two mixed bed size exclusion chromatography columns (Agilent, Mesopore 250 × 4.6 mm, particle diameter 3 µm) with a precolumn (Mesopore 50 × 7.5 mm) operating at 30 °C. THF at a flow rate of 0.30 $mL \cdot min^{-1}$ was used as eluent. The mass spectrometer was coupled to the column in parallel to an UV-detector (VWD 3400, Dionex), and a RI-detector (RefractoMax520, ERC, Japan) in a setup described earlier. 0.27 $mL \cdot min^{-1}$ of the eluent were directed through the UV- and RI-detector and 30 $\mu L \cdot min^{-1}$ were infused into the electrospray source after post-column addition of a 50 µM solution of sodium iodide in methanol at 20 $\mu L \cdot min^{-1}$ by a micro-flow HPLC syringe pump (Teledyne ISCO, Model 100DM). A 200 µL aliquot of a polymer solution with a concentration of 2 $mg \cdot mL^{-1}$ was injected into the SEC system.

1.5 Size exclusion Chromatography THF-GPC

The SEC measurements were conducted on a PSS SECurity2 system consisting of a PSS SECurity Degasser, PSS SECurity TCC6000 Column Oven (35 °C), PSS SDV Column Set (8x150 mm 5 µm Precolumn, 8x300 mm 5 µm Analytical Columns, 100000 Å, 1000 Å and 100 Å) and an Agilent 1260 Infinity Isocratic Pump, Agilent 1260 Infinity Standard Autosampler, Agilent 1260 Infinity Diode Array and Multiple Wavelength Detector

(A: 254 nm, B: 360 nm), Agilent 1260 Infinity Refractive Index Detector (35 °C). HPLC grade THF, stabilized with BHT, is used as eluent at a flow rate of 1 mL·min^{-1}. Narrow disperse linear poly(styrene) (M_n 266 g·mol^{-1} to 2.52·106 g·mol^{-1}) (PSS ReadyCal) was used as calibrants. All samples were passed over 0.22 µm PTFE membrane filters. Molecular weight and dispersity analysis was performed in PSS WinGPC UniChrom software (version 8.2).

1.6 Shimadzu UV-VIS

UV-Vis spectra were recorded on a *Shimadzu* UV-2700 spectrophotometer equipped with a CPS-100 electronic temperature control cell positioner. For molar absorptivity measurements, stock solutions of the samples were prepared in chloroform aliquots were added to a 2 mL solution of chloroform in Thorlabs UV Fused Quartz Cuvettes (CV10Q35F) to obtain five measurements of absorbance between 0 and 1 absorbance units. Spectra were measured at 25 °C.

1.7 LED Characterization

LED emission spectra were recorded using an Ocean Insight Flame-T-UV-Vis spectrometer, with an active range of 200-850 nm and an integration time of 10 ms. LED output energies were recorded using a Thorlabs S401C thermopile sensor, with an active area of 100 mm^2 and a wavelength range of 190 nm – 20 µm, connected to a Thorlabs PM400 energy meter console. The emitted power from each LED was measured for 60 seconds at a fixed distance from the sensor, after which the mean and standard deviation of the emission could be determined. LEDs were cooled during measurement to minimise any thermal effects on the emission power or sensor performance.

1.8 Experiments using LED lamps

The LED-experiments were conducted in a batch setup using a 1.5 mL crimp vial. The ketene (**K1**, 32 µmol, 2.0 eq) and the azobenzene (*trans*-**A1**, 16 µmol, 1.0 eq) were dissolved in dichloromethane (1.5 mL, 15 mmol/L) and irradiated for 10-30 min simultaneously with 385 nm (1 A, 20 V, 2 cm distance) from one side 625 nm (2.1 A, 22 V, 2 cm distance) from the other side. The LEDs were cooled using a stream of air and a fan. After completion the solvent was removed under reduced pressure and submitted to reverse phase HPLC (Acetonitrile:Water gradient from 5:95 to 100:0).

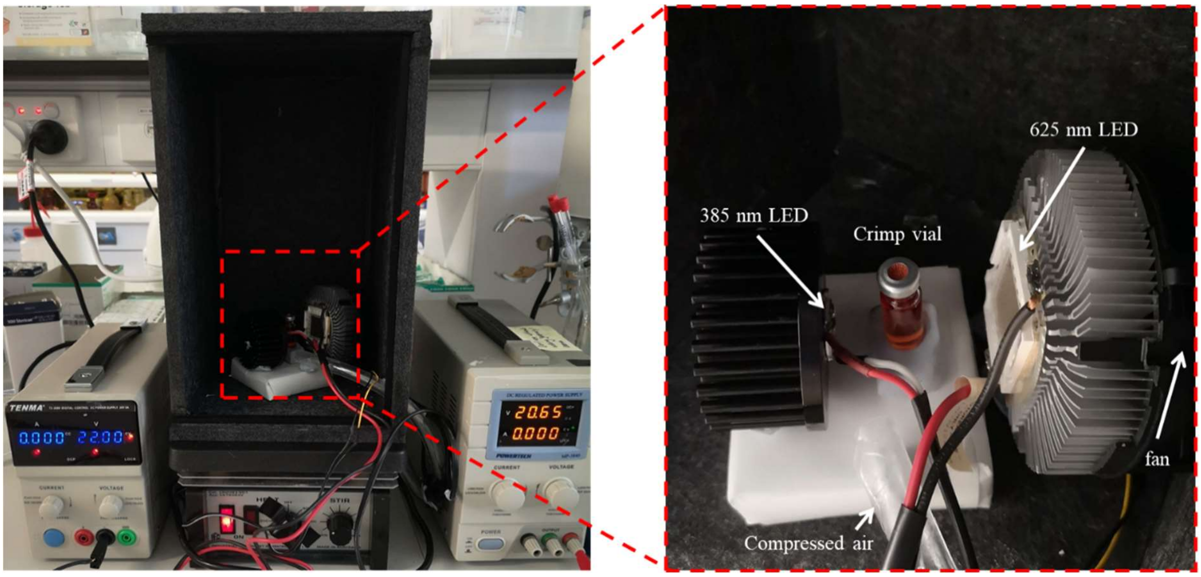

Figure 1 Setup for the irradiation of 1.5 mL crimp-cap *via*ls for the batch experiments. The LEDs and the reaction mixture were cooled by a fan to maintain ambient temperature.

1.9 In-situ Absorbance

The on-line tracking of absorbance spectra during irradiation was conducted using an in-situ UV-Vis apparatus depicted in Figure **S1**. An Ocean Optics DH-MINI Deuterium-Tungsten-Halogen lamp was coupled via optic fibres (P400-025-SR) to an Ocean Optics FLAME-T-UV-VIS spectrometer, sensitive from 200 to 850 nm, via a cuvette holder.

The cuvette holder was situated on a temperature-controlled stage to facilitate temperature dependent switching experiments. An Opolette 355 tuneable OPO, emitting 5 ns pulses from 210-2400 nm at a repetition rate of 20 Hz, was directed onto the side of a quartz fluorescence (Hellma Analytics quartz high precision cell) cuvette perpendicular to the UV-Vis apparatus. Azobenzene *trans*-**A1** samples were prepared in DCM with a concentration of 143 µmol L^{-1} at 25 °C unless noted otherwise and photoreversible ketene (**K3**) samples were prepared in chloroform with a concentration of 52 µmol L^{-1}. Spectra were recorded every 10 s (50 ms integration time, 5 scan average) and processed in Matlab®.

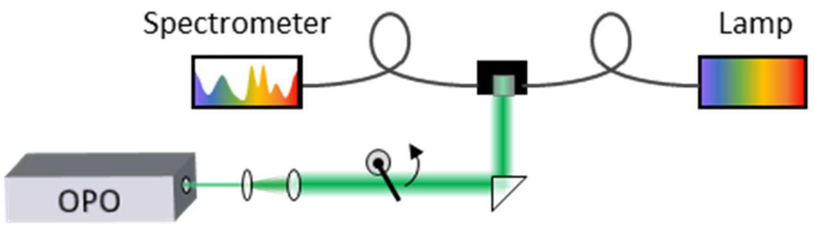

Figure 2 Schematic diagram of experimental apparatus for the photophysical studies. The tuneable OPO output is passed through beam expansion optics before being incident on the side of a quartz fluorescence cuvette. Perpendicular, a UV-Vis lamp is passed through the cuvette and the absorbance spectrum is captured on a spectrometer.

1.10 Kinetics and Action Plot with Opolette Laser

All laser experiments were conducted using the apparatus shown in **Figure S2**. The light source was an Opotek Opolette 355 OPO, producing 7 ns, 20 Hz pulses with a flattop spatial profile. The output beam was initially passed through a beam expander (-50 mm and 100 mm lens combination) to ensure it is large enough to uniformly irradiate the entire sample volume. The beam then passes through an electronic shutter and is directed upwards using a UV silica right angle prism. Finally, the beam enters the sample, suspended in an aluminum block, from below. The laser energy deposited into the sample was measured above the aluminum block before and after experiments using a Coherent EnergyMax thermopile sensor (J-25MB-LE) to account for any power fluctuations during irradiation.

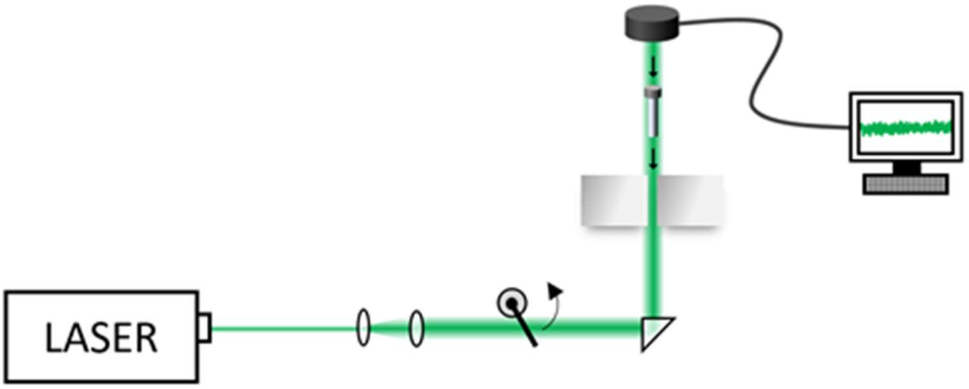

Figure 3 Schematic diagram of apparatus used for laser experiments.

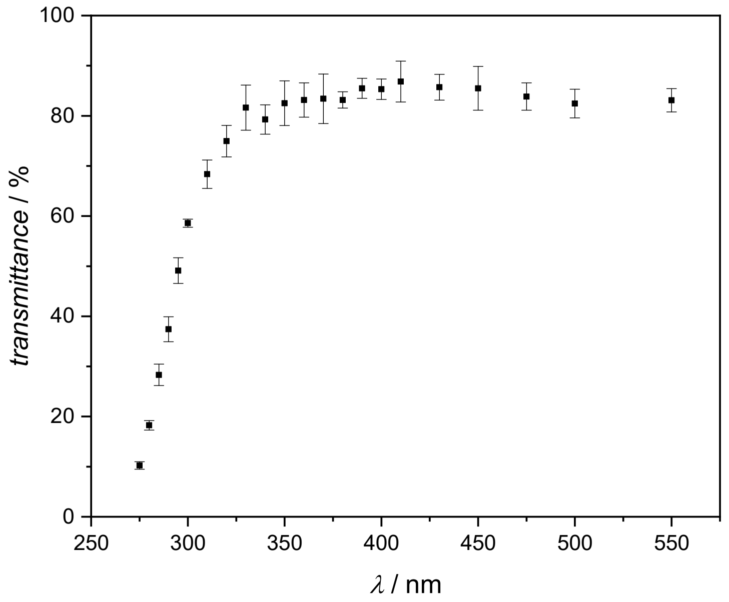

Figure 4 Transmittance of the bottom of the glass vials used in this study. The transmittance values shown and used here were obtained analogously to a method reported previously. The glass vials were cut at a height of 3 mm. Error bars indicate standard deviation in triplicate measurements.

For laser measurements, all samples were prepared in a 0.7 mL glass crimp vials (ID 6.0 mm) capped with a rubber/PTFE septum. The wavelength dependent glass transmittance, essential for quantitative measurements, is presented in **Figure 4**. Precise photons numbers were determined from the laser pulse energy using the following relation

$$N_p = \frac{E_{pulse}\lambda f_{rep} t}{hc\ [T_\lambda/100]} \tag{1}$$

where E_{pulse} is the measured pulse energy above the aluminum block, λ is the wavelength of the incident radiation, f_{rep} is the laser repetition rate, t is the irradiation time, h is Planck's constant, c is the speed of light and T_λ is the wavelength dependent glass transmission presented in **Figure 4**. Once an initial measurement is completed and the photon number is known, the required energies at other wavelengths can be found by rearranging Equation 1 to give

$$E_{pulse} = \frac{N_p hc[T_\lambda/100]}{\lambda f_{rep} t} \tag{2}$$

1.11 Two Color NMR Studies

Two colour NMR experiments were conducted using the apparatus pictured in **Figure 5**. Samples of *trans*-**A1** and **K1** (1.3 mmol L^{-1}, 1:2.3 equiv.) were prepared in CD_2Cl_2 and 0.6 mL was placed in an NMR tube for irradiation. The 385 nm UV light was produced from an Opotek Opolette 355 OPO, producing, 7 ns, 20 Hz pulses with a flattop spatial profile. The output beam was initially passed through a beam expander (-50 mm and 100 mm lens combination) to ensure it is large enough to uniformly irradiate the entire sample volume. The beam subsequently passes through an electronic shutter controlled by an Arduino board and is directed upwards using a UV silica right angle prism. The pulse energy was determined to be (420 ± 20) μJ at the sample holder, measured using a Coherent EnergyMax thermopile sensor (J-25MB-LE). The visible light irradiation was produced by a 10W LED (λ_{max} = 650 nm) mounted 3 cm from the side of the NMR tube. The irradiation time was controlled by an external shutter regulated with an Arduino-board. ^{1}H-NMR spectra were recorded after 60 minutes of irradiation. For the measurement with 385 and 650 nm, the sample was left in the dark for 8 hours after irradiation to achieve maximum product formation.

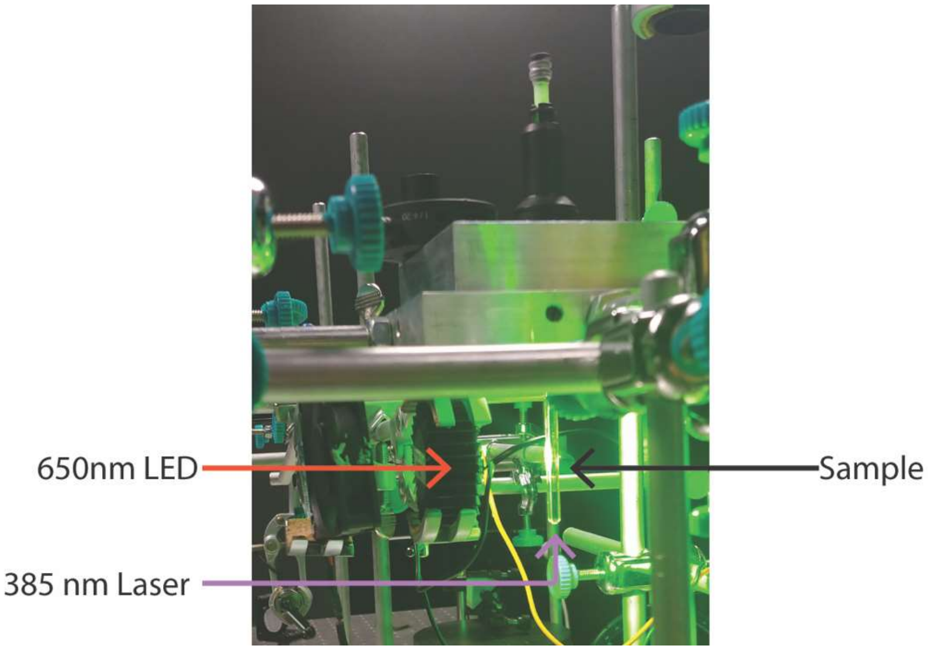

Figure 5 Photograph depicting apparatus for two colour NMR experiments. 385 nm irradiation was delivered via a tuneable OPO directed upwards from below. Visible light LED irradiation is delivered from a 10 W 650 nm LED directed at the sample from the side.

1.12 Rheology Measurements

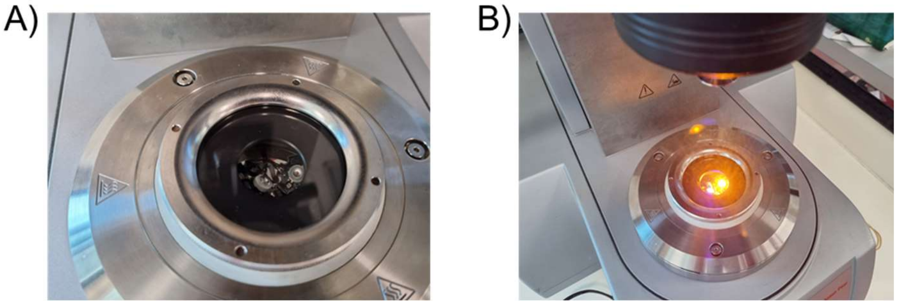

Figure S6 Picture of photo-rheometer setup for 2-colour crosslinking with A) the LED lights placed underneath the quartz plate and B) 2 lights were turned on.

2 Synthesis

2.1 Azobenzene A1

Synthesis of (*E*)-1,2-bis(2,6-dichlorophenyl)diazene

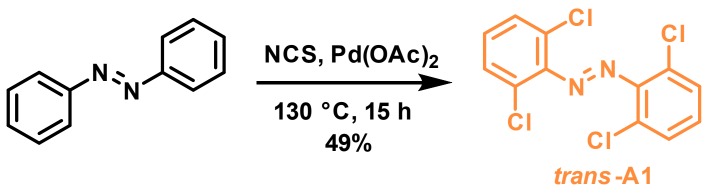

Azobenzene (100 mg, 0.55 mmol, 1.0 eq), NCS (366 mg, 2.74 mmol, 5.0 eq) and palladium acetate (12 mg, 0.055 mmol, 0.1 eq) were dissolved in 5.5 mL AcOH. The round bottom flask was degassed with N_2 and heat up to 130 °C for 15 h. The reaction was cooled to room temperature and the solvent was removed under reduced pressure. The red suspension was extracted with dichloromethane and washed with water (2x100 mL) and brine. The organic layer was dried over $MgSO_4$ and the solvent was removed under reduced pressure. The crude product was submitted to flash chromatography and the product was obtained as a red solid (86 mg, 49%). The NMR spectral data are similar to a previously published report.[1]

^{1}H NMR (600 MHz, Chloroform-*d*) δ 7.46 (d, *J* = 8.1 Hz, 4H), 7.28 – 7.24 (m, 2H).

^{13}C NMR (151 MHz, Chloroform-*d*) δ 147.85, 129.76, 129.56, 127.45.

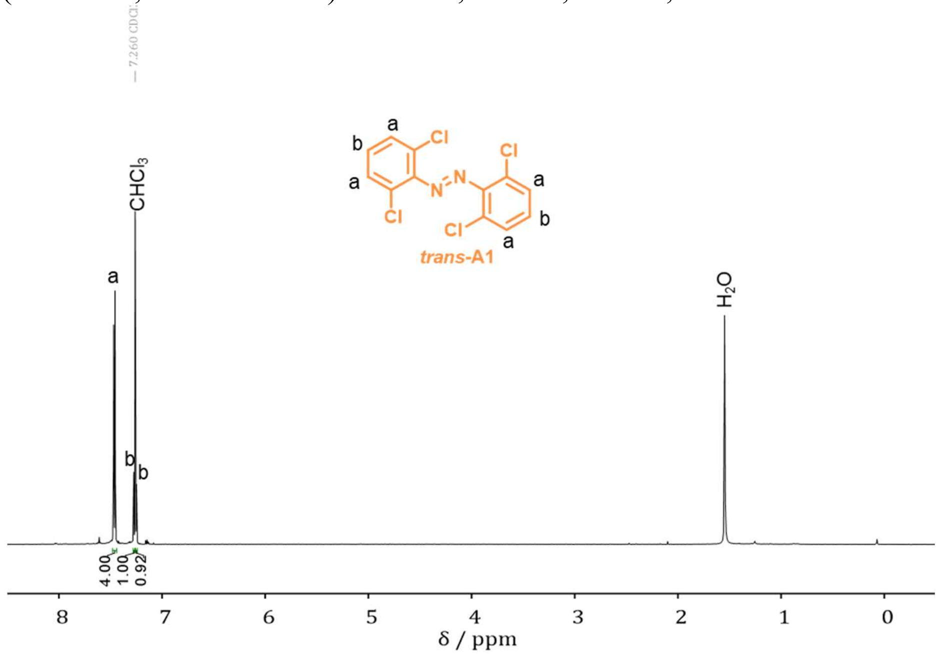

Figure 7 ^{1}H NMR spectrum of (E)-1,2-bis(2,6-dichlorophenyl)diazene (**A1**) recorded in $CDCl_3$ and assigned resonances.

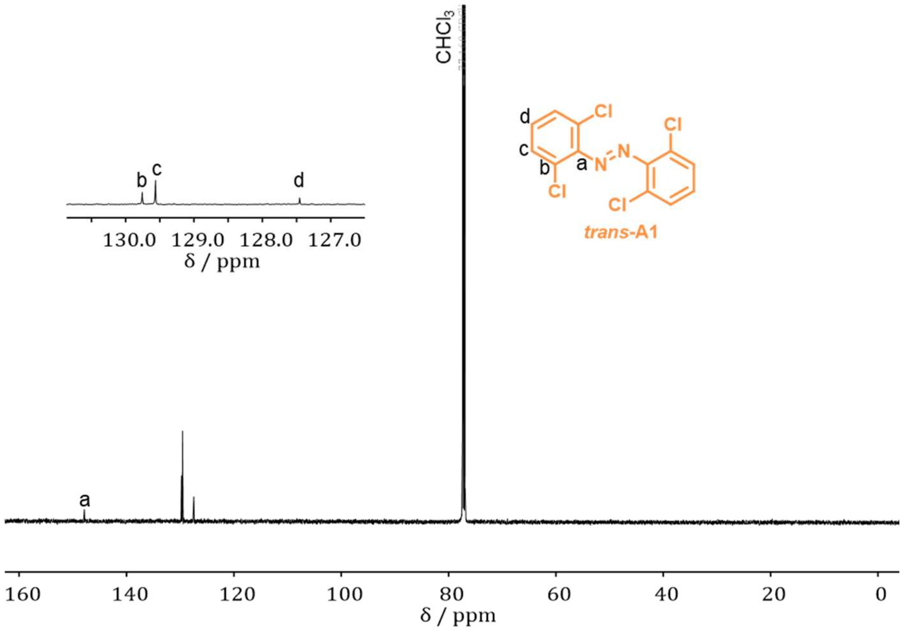

Figure 8 ^{13}C NMR spectrum of (E)-1,2-bis(2,6-dichlorophenyl)diazene (**A1**) recorded in $CDCl_3$ and assigned resonances.

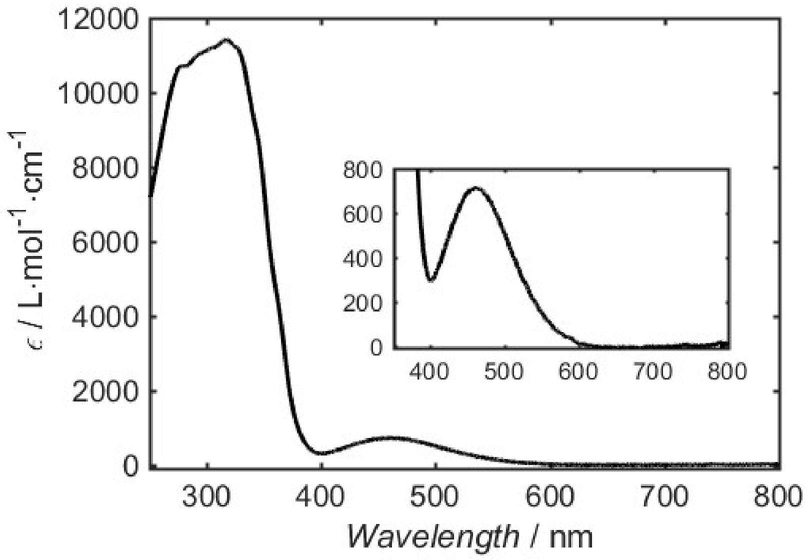

Figure 9 Molar Absorptivity of (E)-1,2-bis(2,6-dichlorophenyl)diazene (**A1**) recorded in chloroform after solvent baseline correction.

2.2 Azobenzene A2, A5

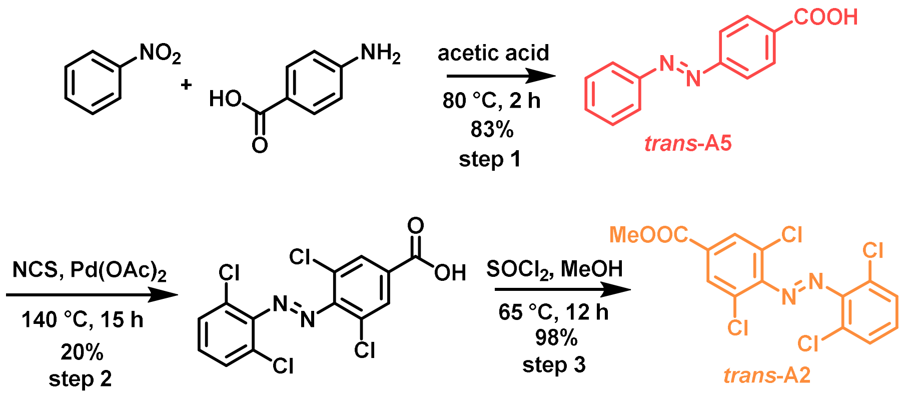

Step 1: methyl (E)-4-(phenyldiazenyl)benzoic acid **A5**

p-aminobenzoic acid (5.4 g, 0.039 mol) was dissolved in glacial acetic acid (40 mL) at 60 ℃ in an Erlenmeyer flask. The solution was cooled to ambient temperature and nitrosobenzene (4.2 g, 0.039 mol) was added under vigorous stirring until a clear solution was obtained. The stirring was stopped and the product begins to crystallize after *ca.* 15 minutes. The crystals were collected by suction filtration and washed with acetic acid (20 mL), water (200 mL), dried in a vacuum oven and used directly in the next step (yield: 7.3 g, 83%). The NMR spectral data are similar to a previously published report.[2]

Step 2: (E)-3,5-dichloro-4-((2,6-dichlorophenyl)diazenyl)benzoic acid

4-(Phenylazo)benzoic acid (1.13 g, 50 mmol), N-chlorosuccinimide (3.325 g, 0.25 mol), $Pd(OAc)_2$ (112 mg, 5 mmol) were dissolved in acetic acid (50 mL) and the solution was purged with nitrogen for 10 min. The reaction vessel was sealed and heated at 135 ℃ for 14 h. The solution was cooled to room temperature and acetic acid was evaporated in vacuo. The crude product was dissolved in CH_2Cl_2 (100 mL) and washed with water (100 mL), brined (100 mL), and dried ($MgSO_4$). The crude product was adsorbed onto silica gel and purified by column chromatography eluting with CH_2Cl_2 : AcOH (v/v = 100/1) to give product as yellow crystal (Yield: 469 mg, 20.2%) that was used directly in the next step. The NMR spectral data are similar to a previously published report.[3]

^{1}H NMR (600 MHz, Chloroform-*d*) δ 8.17 (d, *J* = 0.8 Hz, 2H), 7.49 (dd, *J* = 8.1, 0.8 Hz, 2H), 7.32 – 7.28 (m, 1H).

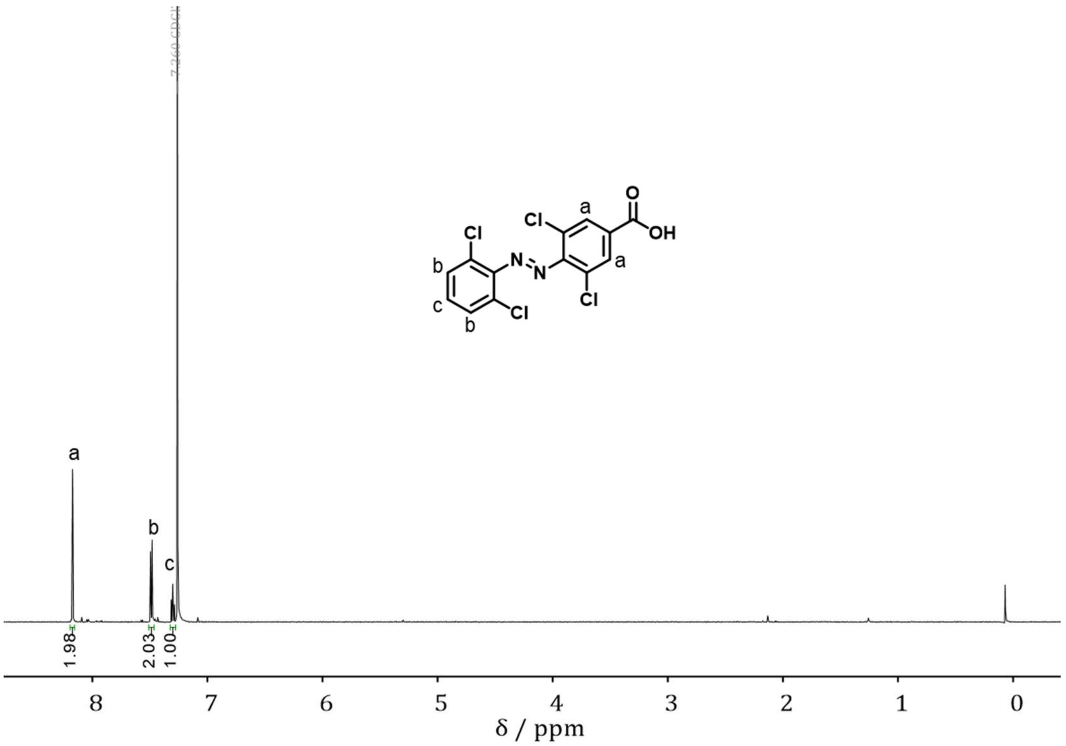

Figure 10 ^{1}H NMR spectrum of (E)-3,5-dichloro-4-((2,6-dichlorophenyl)diazenyl)benzoic acid recorded in $CDCl_3$ and assigned resonances.

Step 3: methyl (E)-3,5-dichloro-4-((2,6-dichlorophenyl)diazenyl)benzoate **A2**

Tetrachloro-azobenzene (150 mg, 0.4 mmol, 1.0 eq) was dissolved in 10 mL of MeOH and thionylchloride (1 mL, excess) was added. The solution was heated at 80 °C under reflux and monitored with TLC (eluting with CH_2Cl_2) until complete consumption of the starting acid material. The solvent was concentrated in vacuo and the product was purified by column chromatography (Dichloromethane/Methanol, DCM/MeOH = 95:5) to yield 152 mg (98%) of red solid **A2**.

^{1}H NMR (600 MHz, Chloroform-*d*) δ 8.11 (s, 2H), 7.48 (d, *J* = 8.1 Hz, 2H), 7.29 (dd, *J* = 8.4, 7.8 Hz, 1H), 3.97 (s, 3H).

^{13}C NMR (151 MHz, Chloroform-*d*) 165.9, 157.6, 153.3, 129.8, 128.3, 127.2, 126, 135.1, 51.

ESI-HRMS (m/z): calculated for $[C_{14}H_8Cl_4N_2O_2+H]^+$: 378.9383, found 378.9389.

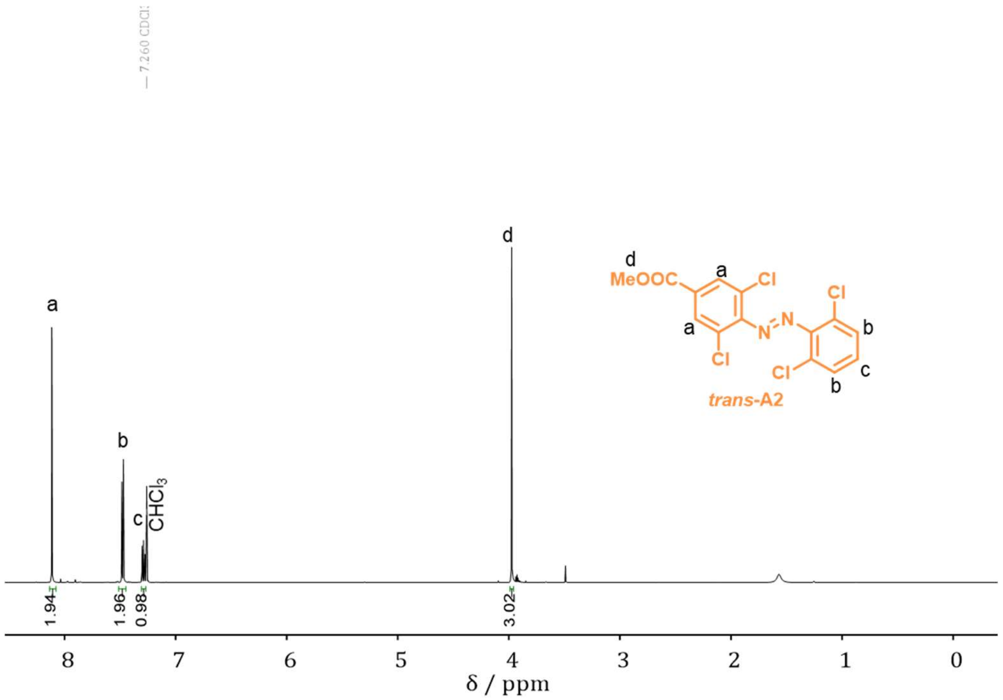

Figure 11 ^{1}H NMR spectrum of ***trans*-A2** recorded in $CDCl_3$ and assigned resonances.

2.3 Photoactive Ketone

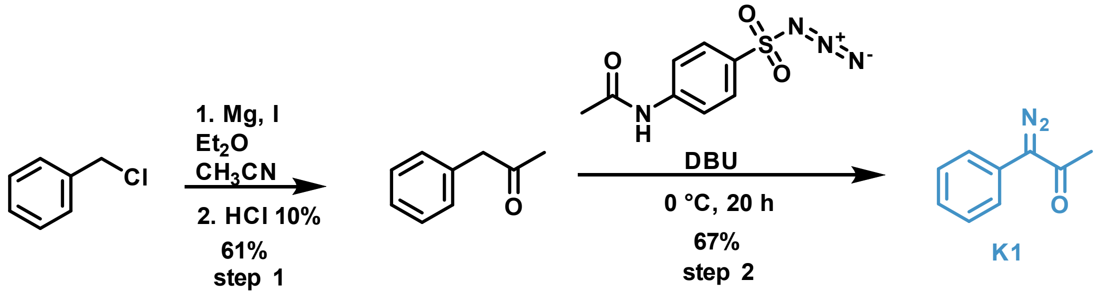

Step 1: 1-phenylpropan-2-one

Benzyl chloride (6.3 g, 0.05 mol) was dissolved in 25 mL Et_2O and cooled on an ice bath. To this solution Mg turnings (1.5 g, 0.0625 mol) were added in portions. Iodine crystals (0.05 g, catalytic mount) were added to initiate the Grignard reaction, followed by a solution of acetonitrile (0.62 g, 0.015 mmol) in 5 mL Et_2O dropwise over 2 h. After completion of the addition, the ice bath was removed and the mixture was heated at 50 °C under refluxing conditions for 2 h. The solution was allowed to cool to ambient temperature and 10% HCl solution (5 mL) was added dropwise under stirring. After all effervescence had stopped, the organic layer was separated and the aqueous phase was washed with Et_2O (50 mL x2). The combined Et_2O layers were washed with H_2O (50 mL x2), dried over MgSO4. The solution

was concentrated in vacuo to give crude phenyl-2-propanone as a clear orange oil, which was used directly in the next step (yield: 4.1 g, 61%). The spectral data are identical to the previously published report.[4]

Step 2: 1-diazo-1-phenylpropan-2-one **K1**

1-Phenylpropan-2-one (4.02 g, 30 mmol) and *p*-acetamidobenzenesulfonyl azide (p-ANSA, 8.3 g, 35 mmol) were dissolved in acetonitrile (50 mL) under a nitrogen blanket and the solution was cooled on an ice bath. A solution of 1,8-diazabicyclo[5.4.0]undec-7-ene (DBU, 5.93 g, 39 mmol) in acetonitrile (10 mL) was added dropwise under stirring. The solution was allowed to warm to ambient temperature and monitored by TLC until complete disappearance of the starting material. The solution was diluted with water (100 mL) and extracted with Et_2O (100 mL x2). The organic layers were combined, concentrated, purified by a flash chromatography on silica gel (hexane/ethyl acetate, PE/EA = 6:1) to give product as pale yellow solid (yield: 3.24 g, 67%). The spectral data are identical to the previously published report.[5]

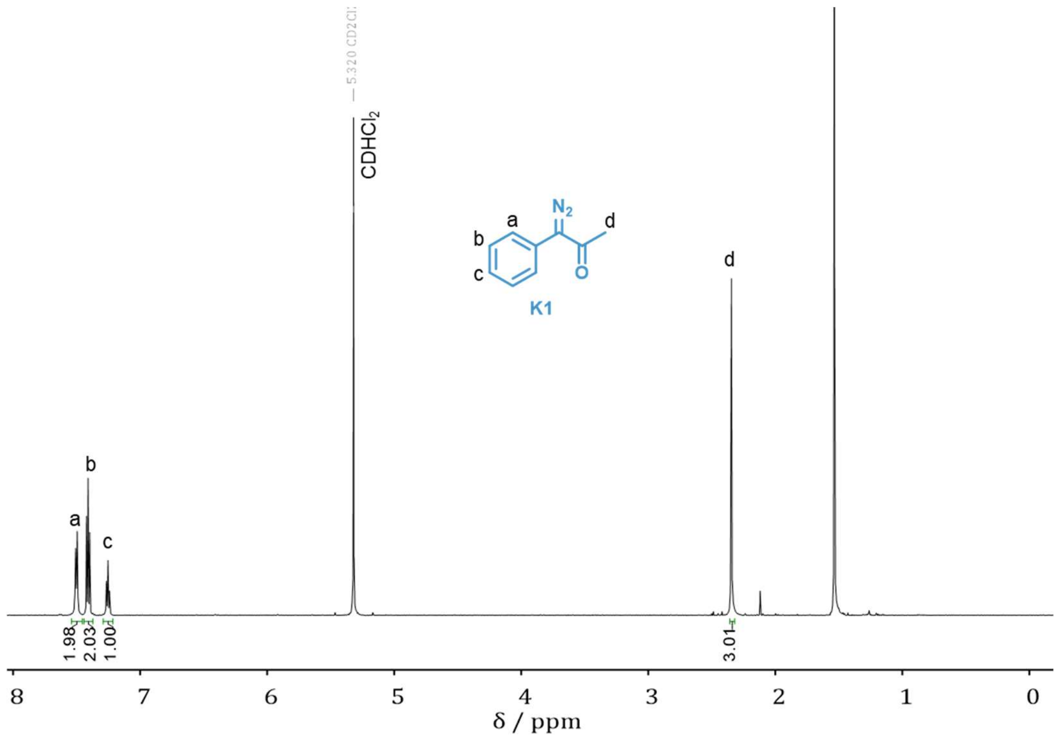

Figure 12 ^{1}H NMR spectrum of 1-diazo-1-phenylpropan-2-one **K1** recorded in CD_2Cl_2 and assigned resonances.

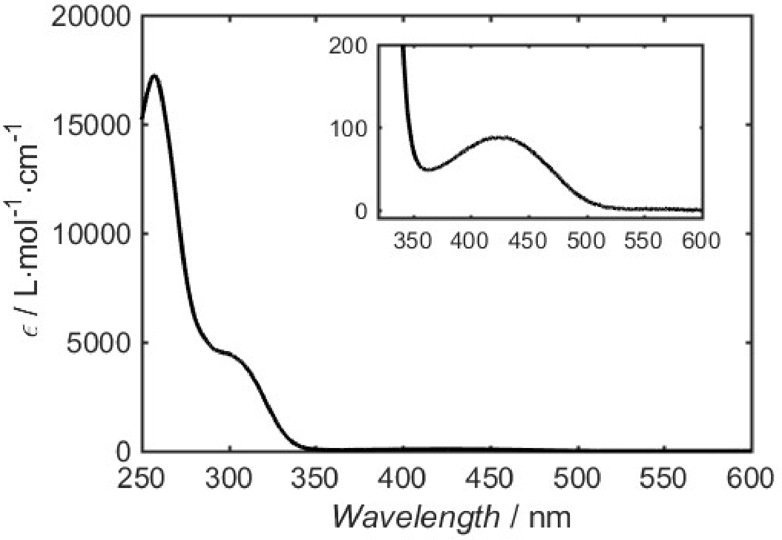

Figure 13 Molar Absorptivity of 1-diazo-1-phenylpropan-2-one (**K1**) recorded in chloroform after solvent baseline correction.

2.4 Photoreversible Ketene (K3)

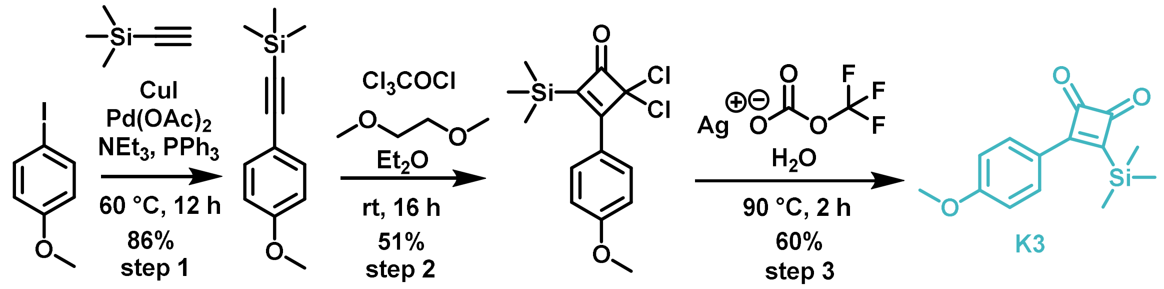

Step 1: ((4-methoxyphenyl)ethynyl)trimethylsilane

4-iodoanisole (2.34 g, 0.01 mol), CuI (41 mg. 0.2 mmol), PPh_3 (111 mg, 0.4 mmol), $Pd(OAc)_2$ (50 mg, 0.2 mmol), and trimethylsilylacetylene (1.18 g, 0.012 mol) were dissolved in trimethylamine (60 mL) and the solution was heated at 80 ℃ under reflux for 12 h. The solution was cooled to ambient temperature, filtered through a Celite layer and washed with diethyl ether. The collected solution was concentrated *in vacuo* to give the product as a yellow oil that was used directly in the next step (yield: 1.75 g, 86%). The spectral data are identical to the previously published report.[6]

Step 2: 4,4-dichloro-3-(4-methoxyphenyl)-2-(trimethylsilyl)cyclobut-2-en-1-one

To a 100 mL flame-dried round bottom flask containing zinc dust (2 g, 0.031 mol) suspension in diethyl ether (20 mL) was added the above alkyne (1.0 g, 5 mmol) and 1,2-dimethoxyethane (2.5 mL). Trichloroacetyl chloride (2 mL, 18 mmol) in diethyl ether (10 mL) was added dropwise under nitrogen over 30 min. The mixture was stirred at ambient temperature for 16 h, filtered, and concentrated in vacuum. The residue was purified by column chromatography running on silica gel and eluting with EtOAc/hexane (v/v = 1/9) to give product as yellow oil (yield 0.803 g, 51%). The spectral data are identical to the previously published report.[7]

^{1}H NMR (600 MHz, Chloroform-*d*) δ 8.06 – 7.85 (m, 2H), 7.14 – 6.95 (m, 2H), 3.92 (s, 3H), 0.38 (s, 9H).

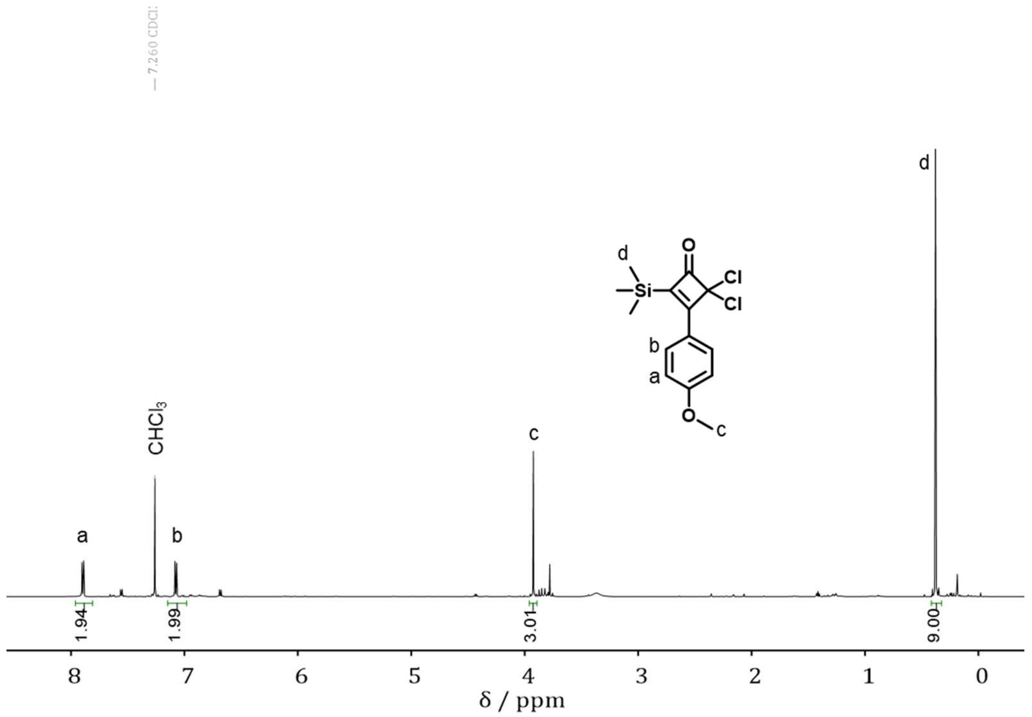

Figure 14 ^{1}H NMR spectrum of 4,4-dichloro-3-(4-methoxyphenyl)-2-(trimethylsilyl) cyclobut-2-en-1-one recorded in $CDCl_3$ and assigned resonances.

Step 3: 3-(4-methoxyphenyl)-4-(trimethylsilyl)cyclobut-3-ene-1,2-dione (**K3**)

The above product (0.5 g, 1.4 mmol) was dissolved in EtOAc (10 mL) and silver trifluoroacetate (1.7 g, 7.7 mmol) was added. The solution was refluxed for 2 h at 90 °C, filtered and water (10 mL) was added to the filtrated. The mixture was stirred vigorously for 1 h and the organic layer was separated, dried with $MgSO_4$, and concentrated in vacuo. The residue was purified by chromatography on silica gel and eluting with hexane to give product as yellow solid (yield: 206 mg, 60%). The spectral data are identical to the previously published report.[8–10]

^{1}H NMR (600 MHz, Chloroform-*d*) δ 8.06 – 7.93 (m, 2H), 7.09 – 7.03 (m, 2H), 3.91 (s, 3H), 0.46 (s, 9H).

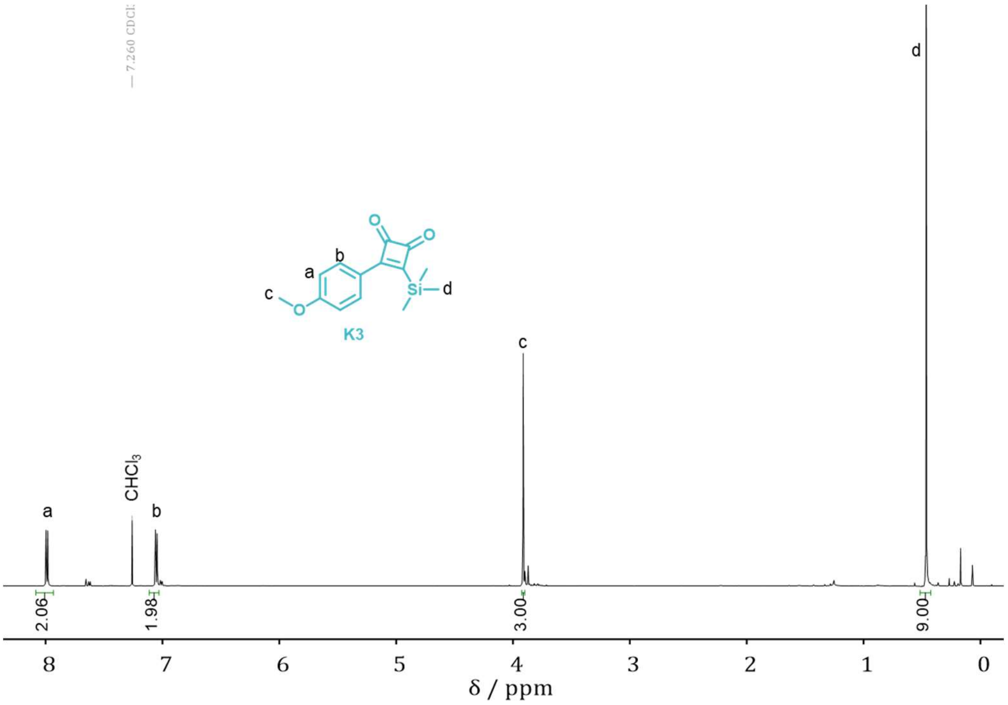

Figure 15 ^{1}H NMR spectrum of 3-(4-methoxyphenyl)-4-(trimethylsilyl)cyclobut-3-ene-1,2-dione **K3** recorded in $CDCl_3$ and assigned resonances.

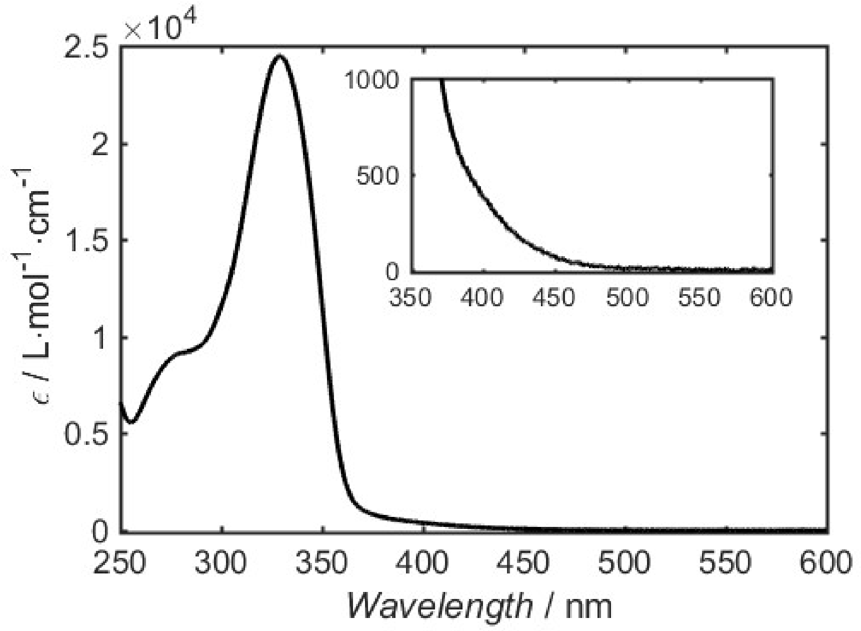

Figure 16 Molar Absorptivity of 3-(4-methoxyphenyl)-4-(trimethylsilyl)cyclobut-3-ene-1,2-dione (**K3**) recorded in chloroform after solvent baseline correction.

2.5 Photoreversible tetra-Ketene (K4)

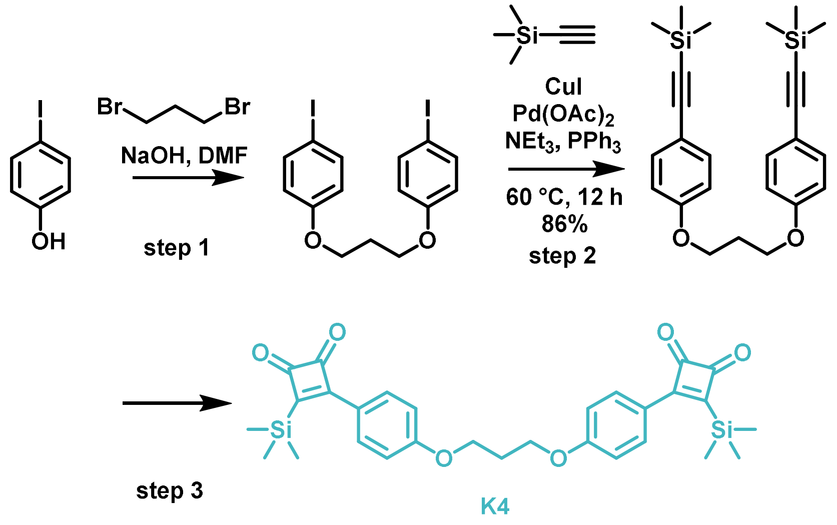

Step 1: 1,3-bis(4-iodophenoxy)propane

1,3-dibromopropane (2g ,10 mmol) and 4-iodophenol (4.84 g, 22 mmol) were dissolved in DMF (20 mL) and a solution of NaOH (2 g, 50 mmol) in water (5 mL) was added. The solution was heated at 80 °C for 4 h and allowed to cool to ambient temperature. The mixture was filtered and the filtrate was concentrated in vacuo. The residue was redissolved in EtOAc (100 mL) and washed with water (50 mL), brine (50 mL), and dried with $MgSO_4$. Partial evaporation of the solution to ca. 30 mL induced formation of crystal and the solution was allowed to stand

at ambient temperature for 2 h during which colourless crystal was formed. The product was filtered and dried in vacuo (yield: 2.74 g, 57%). The spectral data are identical to the previously published report.[11]

^{1}H NMR (600 MHz, Chloroform-*d*) δ 7.62 – 7.36 (m, 4H), 6.82 – 6.54 (m, 4H), 4.10 (t, *J* = 6.1 Hz, 4H), 2.24 (p, *J* = 6.1 Hz, 2H).

^{13}C NMR (151 MHz, Chloroform-*d*) δ 158.82, 138.37, 117.04, 82.98, 64.55, 29.23.

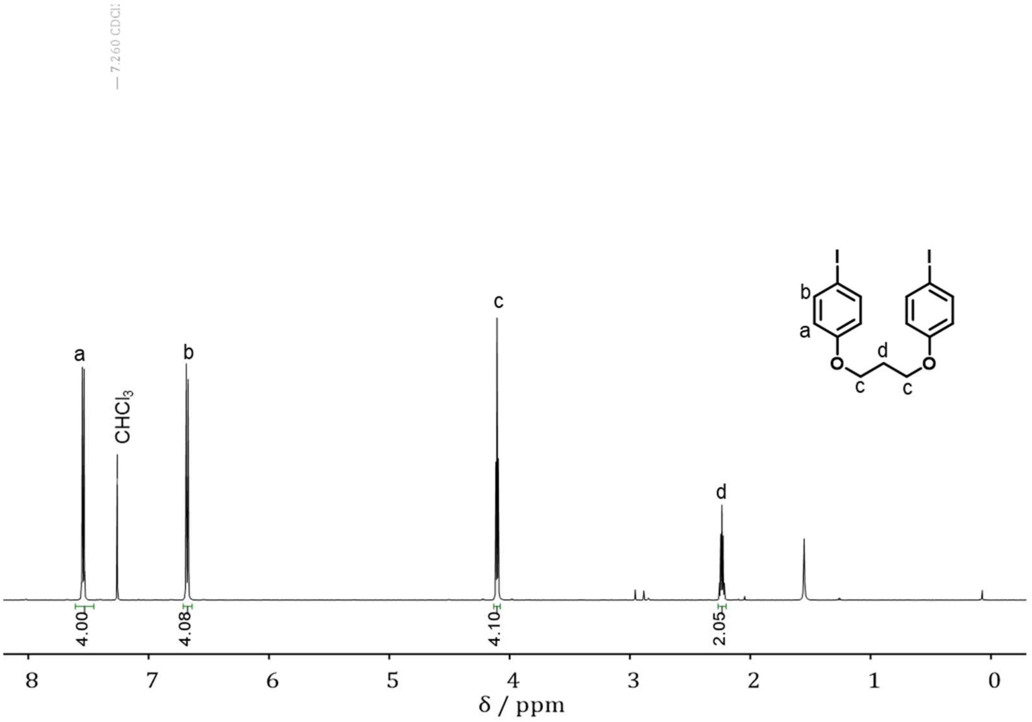

Figure 17 ^{1}H NMR spectrum of 1,3-bis(4-iodophenoxy)propane recorded in $CDCl_3$ and assigned resonances.

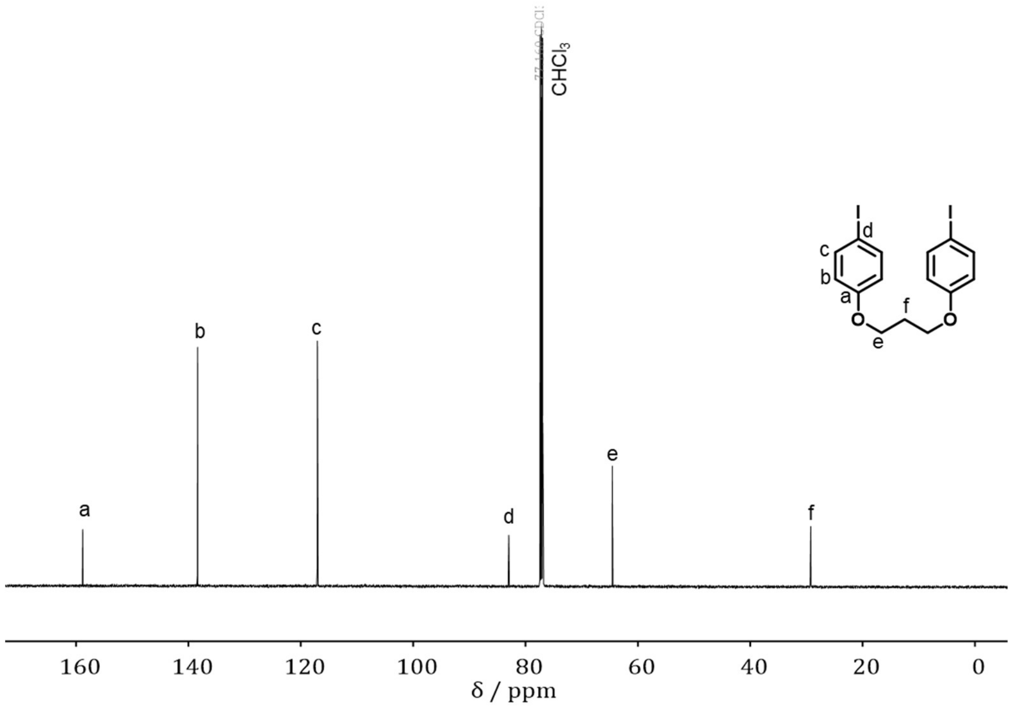

Figure 18 ^{13}C NMR spectrum of 1,3-bis(4-iodophenoxy)propane recorded in $CDCl_3$ and assigned resonances.

Step 2: 1,3-bis(4-((trimethylsilyl)ethynyl)phenoxy)propane

The above product (1.44 g, 3 mmol) was dissolved in NEt_3 (60 mL) and to this solution triphenylphosphine (157 mg, 0.6 mmol), CuI (57 mg, 0.3 mmol), trimethylsilyl acetylene (0.98 g, 10 mmol) and Pd(II) acetate (67.2 mg, 0.3 mmol) was added. The solution was heated to 60 ^{o}C for 16 h. After cooling to ambient temperature, the solution was filtered and concentrated in vacuo. The residue was purified by chromatography on silica gel and eluting with hexane to give product as colourless crystal (yield: 0.983 g, 78%). The spectral data are identical to the previously published report.[6]

^{1}H NMR (600 MHz, Chloroform-*d*) δ 7.43 – 7.36 (m, 4H), 6.90 – 6.69 (m, 4H), 4.14 (t, *J* = 6.1 Hz, 4H), 2.34 – 2.17 (m, 2H), 0.24 (s, 18H).

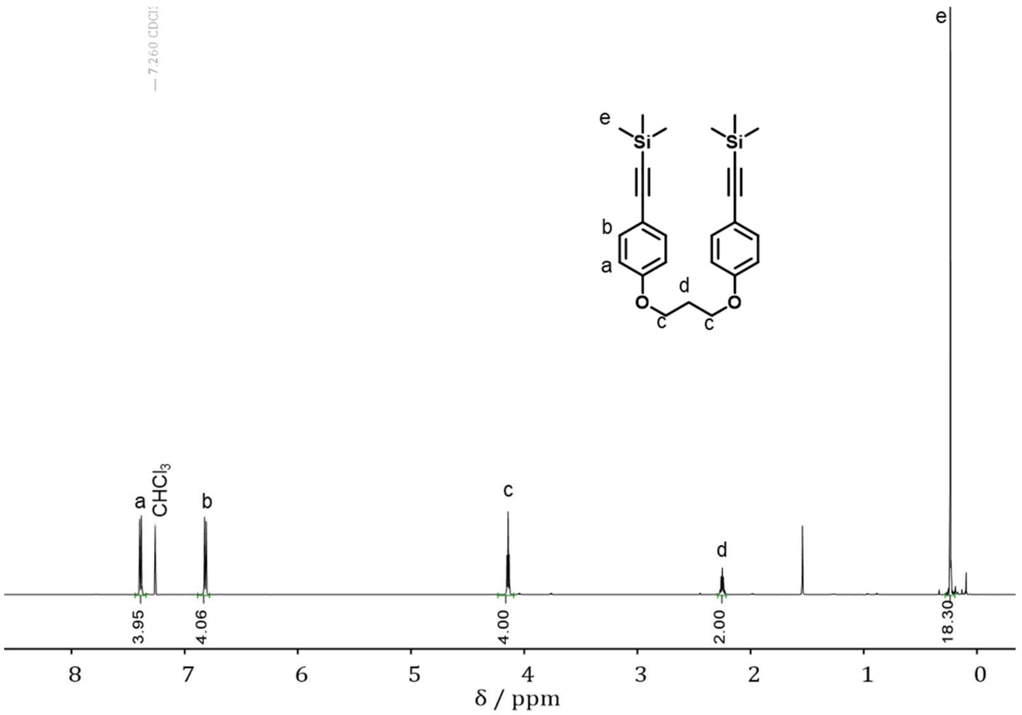

Figure 19 ^{1}H NMR spectrum of 1,3-bis(4-((trimethylsilyl)ethynyl)phenoxy)propane recorded in $CDCl_3$ and assigned resonances.

Step 3: 4,4'-((propane-1,3-diylbis(oxy))bis(4,1-phenylene))bis(3-(trimethylsilyl)cyclobut-3-ene-1,2-dione)

$Fe(CO)_5$ (6 g, 30 mmol) was added dropwise to a solution of anhydrous *t*-BuOK (3.36 g, 30 mmol) in THF (100 mL) at 25 °C under a nitrogen blanket. The solution was heated to 70 ℃ for 1 h during which the solution colour turned from yellow to dark brown. The solution was cooled to ambient temperature and 1,3-bis(4-((trimethylsilyl)ethynyl)phenoxy)propane (0.84 g, 2 mmol) was added. The solution was stirred at 70 ℃ for 14 h. Upon cooling to ambient temperature, $CuCl_2 \cdot 2H_2O$ (10 g, 60 mmol) in acetone (50 mL) was added and the solution was stirred for 1 h at ambient temperature. The solution was filtered, concentrated *in vacuo* and the residue was purified by column chromatography running on silica gel and eluting with EtOAc/hexane (v/v = 1/9) to give product as yellow solid (yield: 0.414 g, 39%).

^{1}H NMR (600 MHz, Chloroform-*d*) δ 8.04 – 7.90 (m, 4H), 7.12 – 7.01 (m, 4H), 4.29 (t, *J* = 6.0 Hz, 4H), 2.37 (t, *J* = 6.0 Hz, 2H), 0.46 (s, 18H).

^{13}C NMR (151 MHz, Chloroform-*d*) δ 132.35, 115.62, 64.90, 2.15.

ESI-HRMS (m/z): calculated for $[C_{29}H_{32}O_6Si_2+NH_4]^+$: 550.2076, found 550.2071.

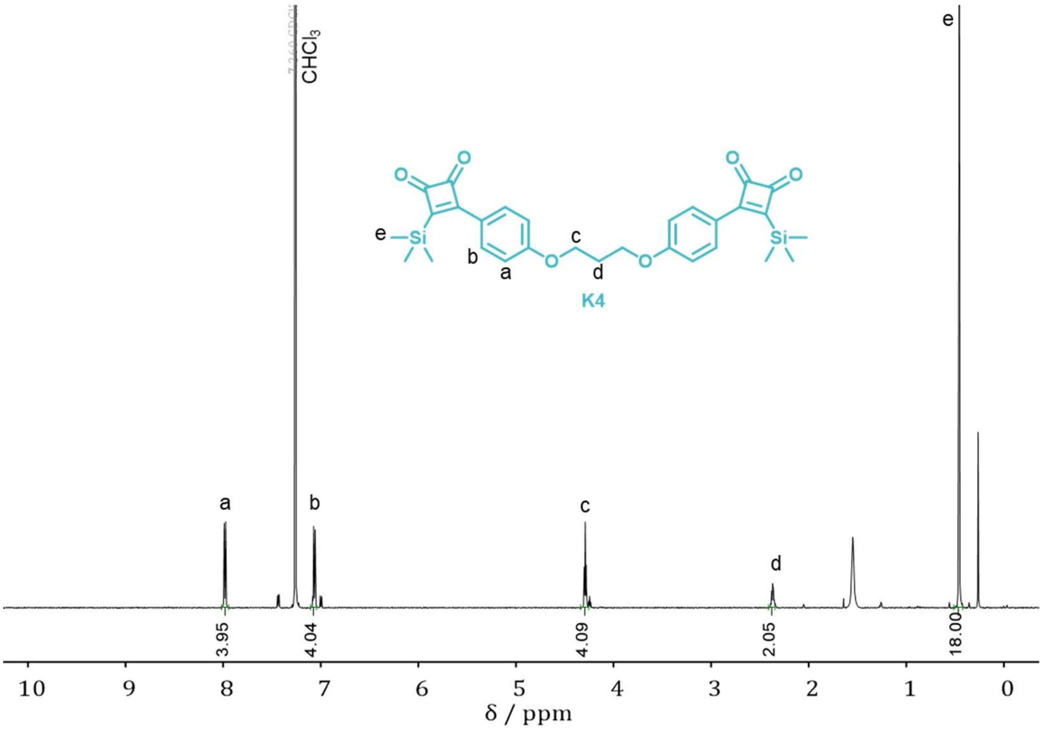

Figure 20 ^1H NMR spectrum of 4,4'-((propane-1,3-diylbis(oxy))bis(4,1-phenylene))bis(3-(trimethylsilyl)cyclobut-3-ene-1,2-dione) (**K4**) recorded in $CDCl_3$ and assigned resonances.

2.6 PEG-(Cl_4-Azobenzene) (A3)

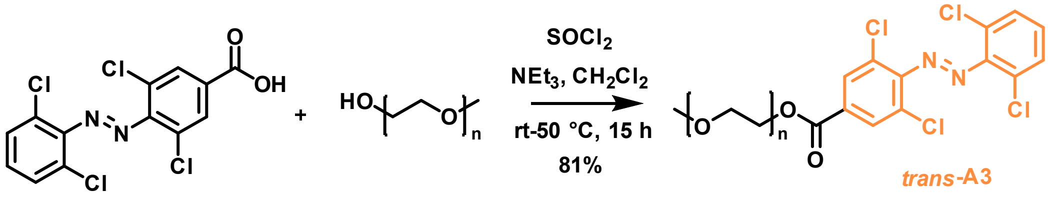

Azobenzene carboxylic acid (436 mg, 1.2 mmol) was dissolved in a solution of dichloromethane (30 mL) and thionyl chloride (2 mL, excess). DMF (20 µL, catalytic amount) was added and the solution was heated under reflux at 50 °C. The reaction was monitored by TLC (running with CH_2Cl_2) until complete disappearance of the starting material. The solution was evaporated in vacuo and co-evaporated twice with CH_2Cl_2 (20 mL). The solution was redissolved in CH_2Cl_2 (10 mL) and MeO-PEG-OH (200 mg, 1 mmol) was added. The solution was cooled on an ice bath and NEt_3 (0.5 g, 5 mmol) was added. The solution was allowed to

warm to ambient temperature and stirred for 15 h, before evaporating *in vacuo* to ca. 2 mL. The solution was precipitated into diethyl ether (100 mL) to give product as red solid (yield: 191 mg, 80.9%).

^{1}H NMR (600 MHz, Chloroform-*d*) δ 8.10 (s, 1H), 7.46 (d, J = 8.1 Hz, 1H), 7.28 (d, J = 8.1 Hz, 1H), 4.54 – 4.47 (m, 1H), 3.86 – 3.80 (m, 1H), 3.71 – 3.47 (m, 177H), 3.35 (s, 3H).

^{13}C NMR (151 MHz, Chloroform-*d*) δ 163.80, 130.45, 130.17, 129.56, 80.36, 76.55, 72.53, 71.93, 70.56, 70.32, 68.96, 64.99, 61.70, 59.02.

Detailed mass spectra available in Figure 56.

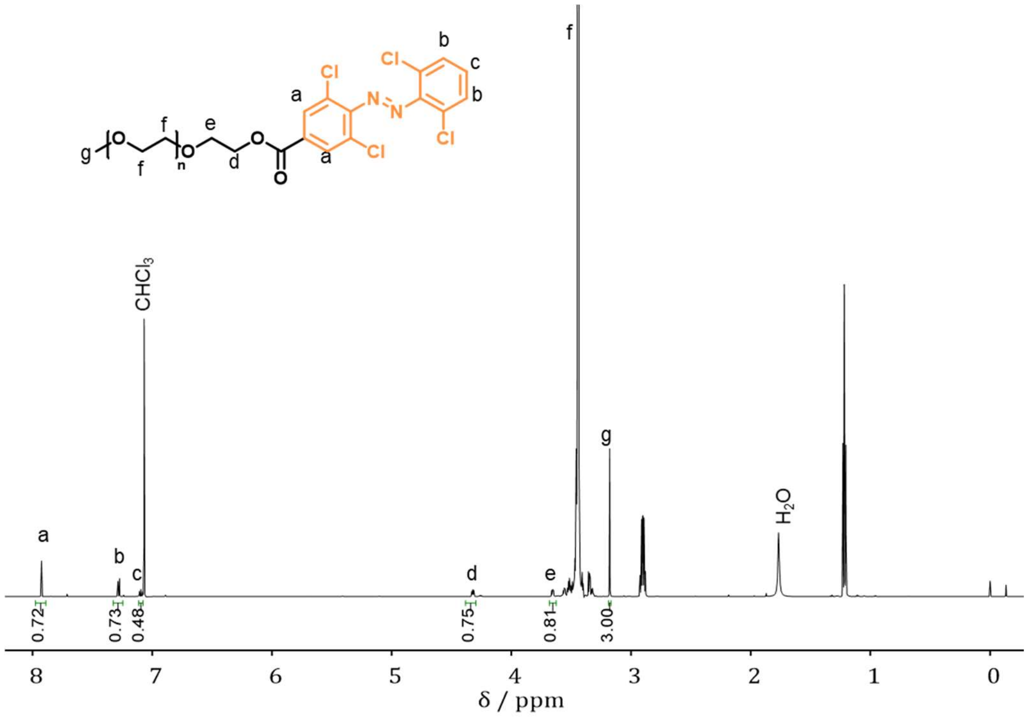

Figure 21 ^{1}H NMR spectrum of **A3** recorded in $CDCl_3$ and assigned resonances.

2.7 PEG-(Cl_4-Azobenzene)$_3$ crosslinker (A4)

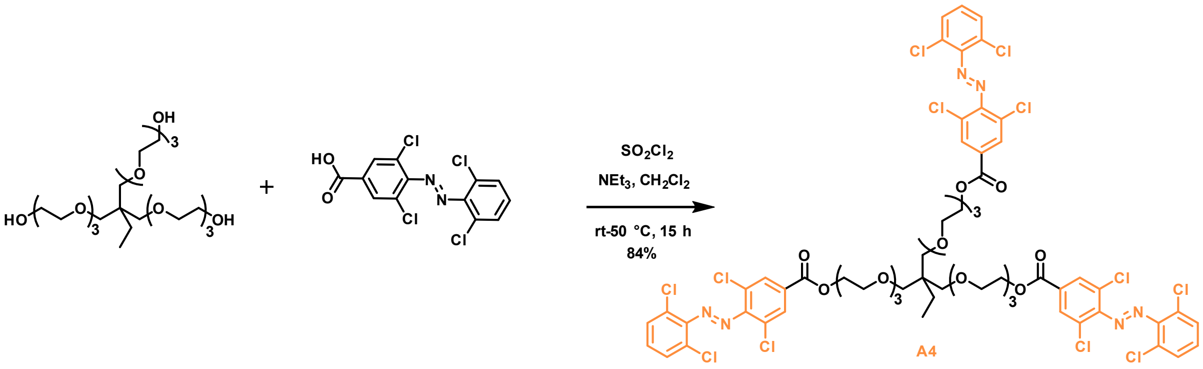

Compound **A2** (600 mg, 1.65 mmol) was dissolved in a solution of dichloromethane (30 mL) and thionyl chloride (2 mL, excess). DMF (20 μL, catalytic amount) was added and the solution was heated under refluxing at 50 °C. The reaction was monitored by TLC (running with CH_2Cl_2) until complete disappearance of the starting material. The solution was evaporated in vacuo and co-evaporated twice with CH_2Cl_2 (20 mL). The solution was redissolved in CH_2Cl_2 (10 mL) and trimethylpropanne ethoxylate (225 mg, 0.5 mmol) was added. The solution was cooled on an ice bath and NEt_3 (0.5 g, 5 mmol) was added. The solution was allowed to warm to ambient temperature and stirred for 15 h, before evaporating *in vacuo*. The residue was purified by column chromatography running on silica gel and eluting with CH_2Cl_2/MeOH (v/v = 10/1) to give product as red oil (yield: 625 mg, 84%).

^{1}H NMR (600 MHz, Chloroform-*d*) δ 8.12 – 8.00 (m, 2H), 7.52 – 7.30 (m, 2H), 7.24 – 7.15 (m, 1H), 4.49 – 4.41 (m, 2H), 3.83 – 3.74 (m, 2H), 3.68 – 3.44 (m, 10H), 3.41 – 3.22 (m, 2H), 1.28 (d, J = 7.5 Hz, 1H), 0.86 – 0.69 (m, 1H).

^{13}C NMR (151 MHz, Chloroform-*d*) δ 164.15, 151.22, 147.57, 131.48, 131.11, 130.83 (d, J = 5.5 Hz), 130.48, 129.90, 128.01, 127.27, 73.65, 72.00 – 70.20 (m), 69.32, 65.33, 31.28, 7.95.

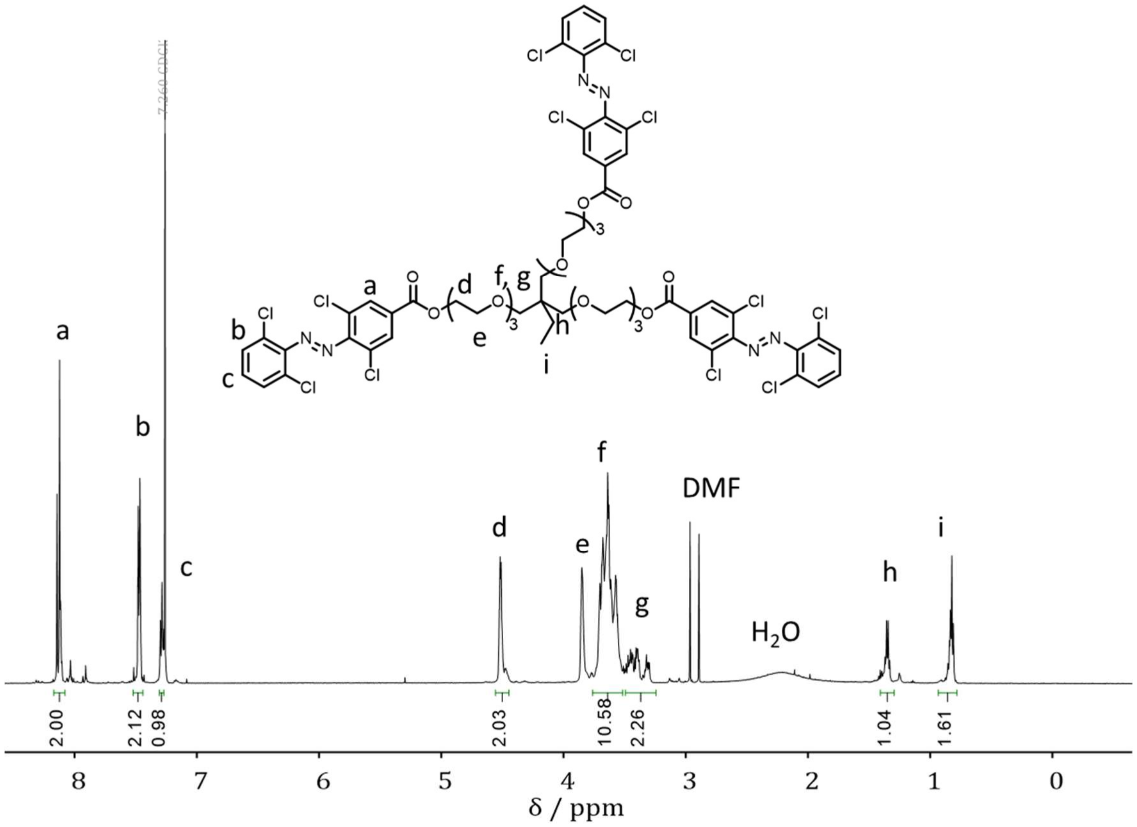

Figure 22 ^{1}H NMR spectrum of **A4** recorded in $CDCl_3$ and assigned resonances.

2.8 Tri(ethylene glycol) Ketene K5

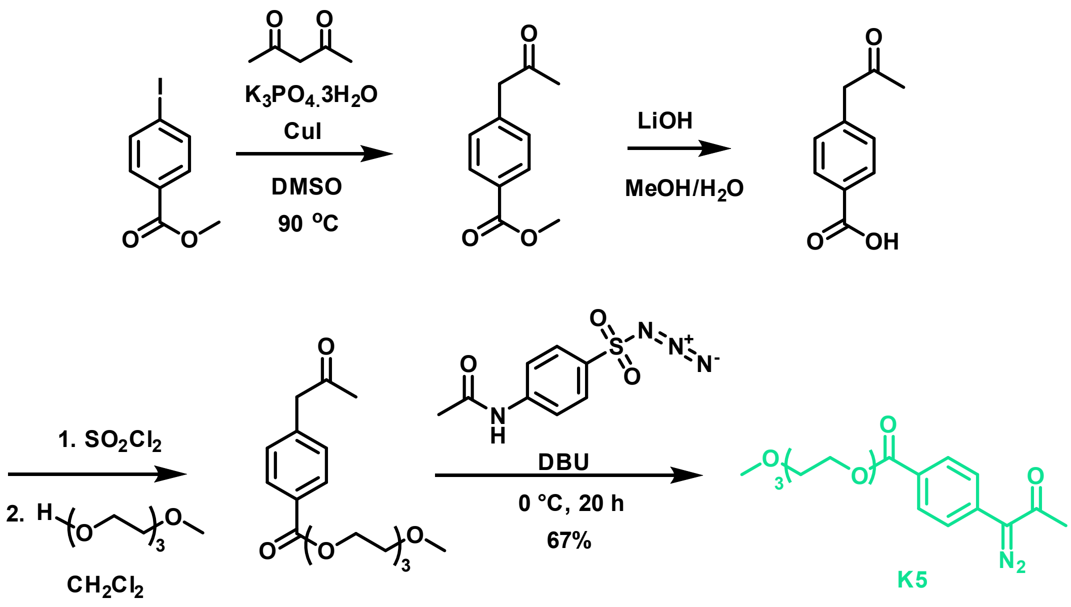

The preparation of the intermediates for K5 was carried according to a previously published procedure and with identical ^{1}H NMR spectral data of all compounds.[12]

Step 1: methyl 4-(2-oxopropyl)benzoate

4-Iodomethylbenzoate (5.24 g, 0.02 mol) and acetylacetone (6 g, 0.06 mol) were dissolved in DMSO (60 mL). To this solution was added CuI (380 mg, 2 mmol), K_3PO_4 (14g, 0.06 mol) and water (3.24g, 0.18 mol). The solution was bubbled with nitrogen gas for 30 min, sealed and stirred at 90 °C for 16 h. The solution was allowed to cool to room temperature, diluted with water (100 mL) and extracted with ethylacetate (100 mL x 3). The combined organic phases were washed with brine (100 mL), dried and purified by column chromatography running on SiO_2, eluting with EtOAC/n-hexane (v/v = 1/3) to give product as yellow liquid (yield: 2.7 g; 71%).

Step 2: 4-(2-oxopropyl)benzoic acid

Methyl 4-(2-oxopropyl)benzoate (1.92 g, 0.01 mol) was dissolved in methanol (10 mL) and LiOH (0.02 mol) in water (5 mL) was added. The solution was stirred at ambient temperature and monitored by TLC (dichloromethane) until complete disappearance of the starting materials. The solution was acidified to pH 2 by slow addition of HCl 2M, and extracted with ethyl acetate (50 mL x 2). The organic phases were dried ($MgSO_4$) and purified by column chromatography running on SiO_2, eluting with CH_2Cl_2/MeOH (v/v = 1/0.02) to give product as pale yellow solid (yield: 1.34 g; 75%).

Step 3: 2-(2-(2-methoxyethoxy)ethoxy)ethyl 4-(2-oxopropyl)benzoate

4-(2-Oxopropyl)benzoic acid (0.89 g, 5 mmol) was dissolved in CH_2Cl_2 (10 mL) and thionyl chloride (1 mL, excess) was added. The solution was heated at 50 °C under refluxing condition and monitored by TLC until complete disappearance of the starting acid material. The solution was concentrated in vacuo and co-evaporated with chloroform (20 mL x 2) to remove the thionylchloride. The residue was re-dissolved in CH_2Cl_2 (10 mL) and methoxytriethylene glycol (0.82 g, 5 mmol) was added. The solution was cooled on an ice bath and NEt_3 (1g, 10 mmol). The solution was stirred at ambient temperature for 16 h and, purified by column chromatography on SiO_2, eluting with CH_2Cl_2 to give product as clear oil (yield: 1.33 g; 83%).

Step 4: 2-(2-(2-methoxyethoxy)ethoxy)ethyl 4-(1-diazo-2-oxopropyl)benzoate

2-(2-(2-methoxyethoxy)ethoxy)ethyl 4-(2-oxopropyl)benzoate (648 mg, 2 mmol) and p-acetamidobenzenesulfonyl azide (2.5 mmol) were dissolved in acetonitrile (10 mL) under a nitrogen blanket and the solution was cooled on an ice bath. A solution of 1,8-diazabicyclo[5.4.0]undec-7-ene (2.7 mmol) in acetonitrile (2 mL) was added dropwise under

stirring. The solution was allowed to warm to ambient temperature and monitored by TLC until complete disappearance of the starting material. The solution was diluted with water (100 mL) and extracted with EtOAC (50 mL x2). The organic layers were combined, concentrated, purified by a flash chromatography on silica gel (hexane/ethyl acetate, PE/EA = 1:5) to give product as pale-yellow oil (yield: 465 mg; 62%).

^{1}H NMR (600 MHz, Chloroform-*d*) δ 8.00 (d, *J* = 8.7 Hz, 2H), 7.54 (d, *J* = 8.6 Hz, 2H), 4.46 – 4.37 (m, 2H), 3.81 – 3.75 (m, 2H), 3.64 (qt, *J* = 3.9, 1.7 Hz, 2H), 3.62 – 3.56 (m, 4H), 3.49 – 3.45 (m, 2H), 3.30 (s, 3H), 2.35 (s, 3H).

^{13}C NMR (151 MHz, Chloroform-*d*) δ 165.98, 130.31, 128.00, 127.65, 124.39, 71.93, 71.18 – 70.23 (m), 69.22, 64.18 (d, *J* = 13.1 Hz), 59.05, 27.22, 18.23.

ESI-HRMS (m/z): calculated for $[C_{17}H_{22}N_2O_6+NH_4]^+$: 368.1816 found 368.1814.

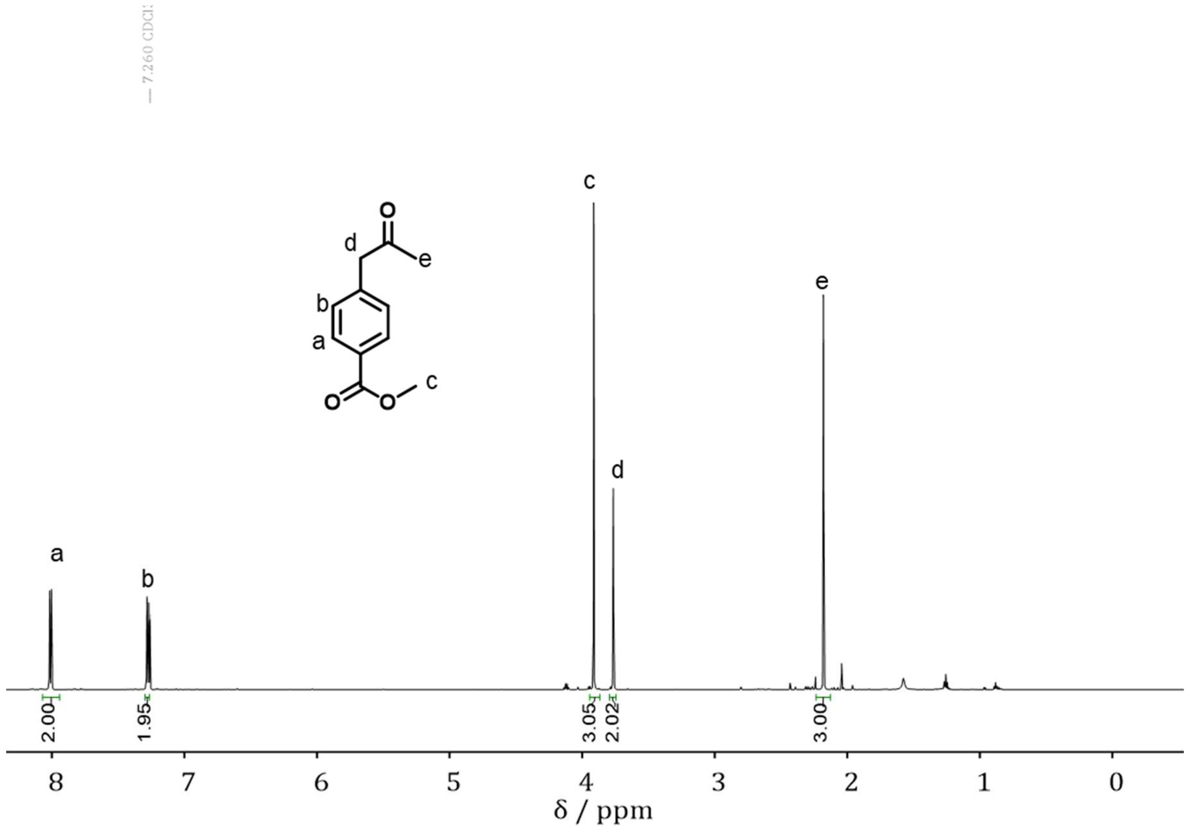

Figure 23 ^{1}H NMR spectrum of methyl 4-(2-oxopropyl)benzoate recorded in $CDCl_3$ and assigned resonances.

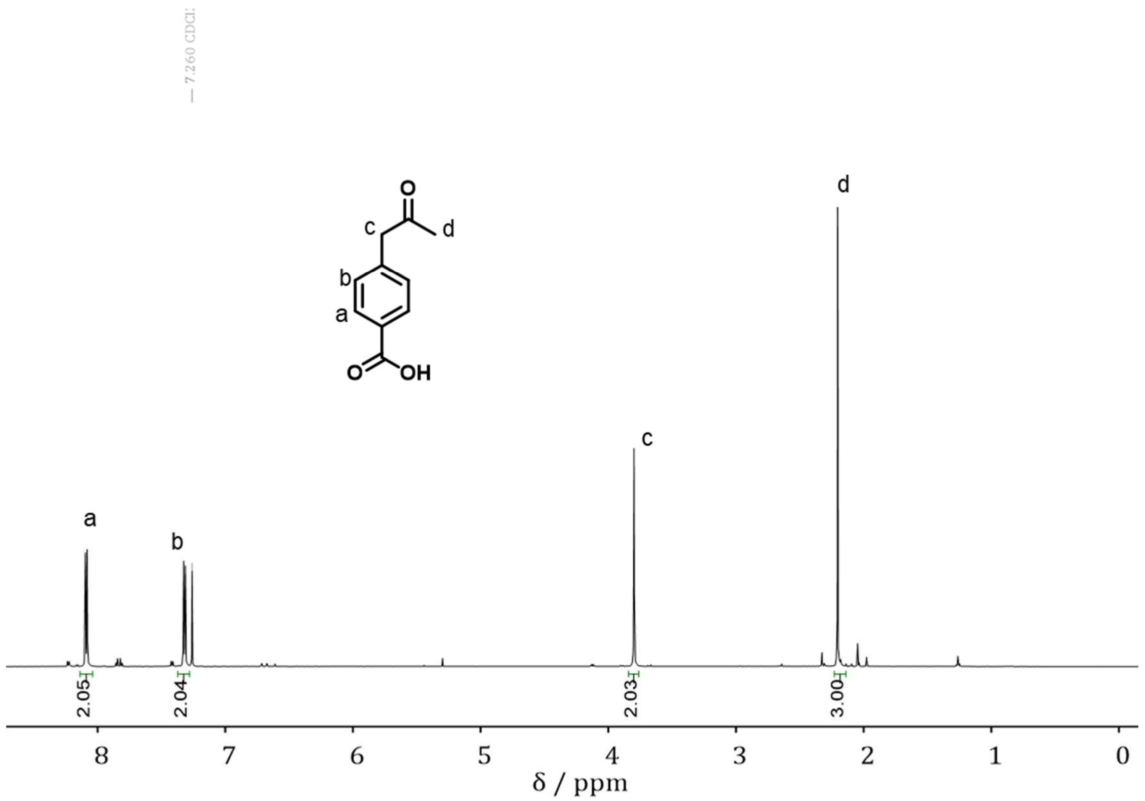

Figure 24 ^{1}H NMR spectrum of 4-(2-oxopropyl)benzoic acid recorded in $CDCl_3$ and assigned resonances.

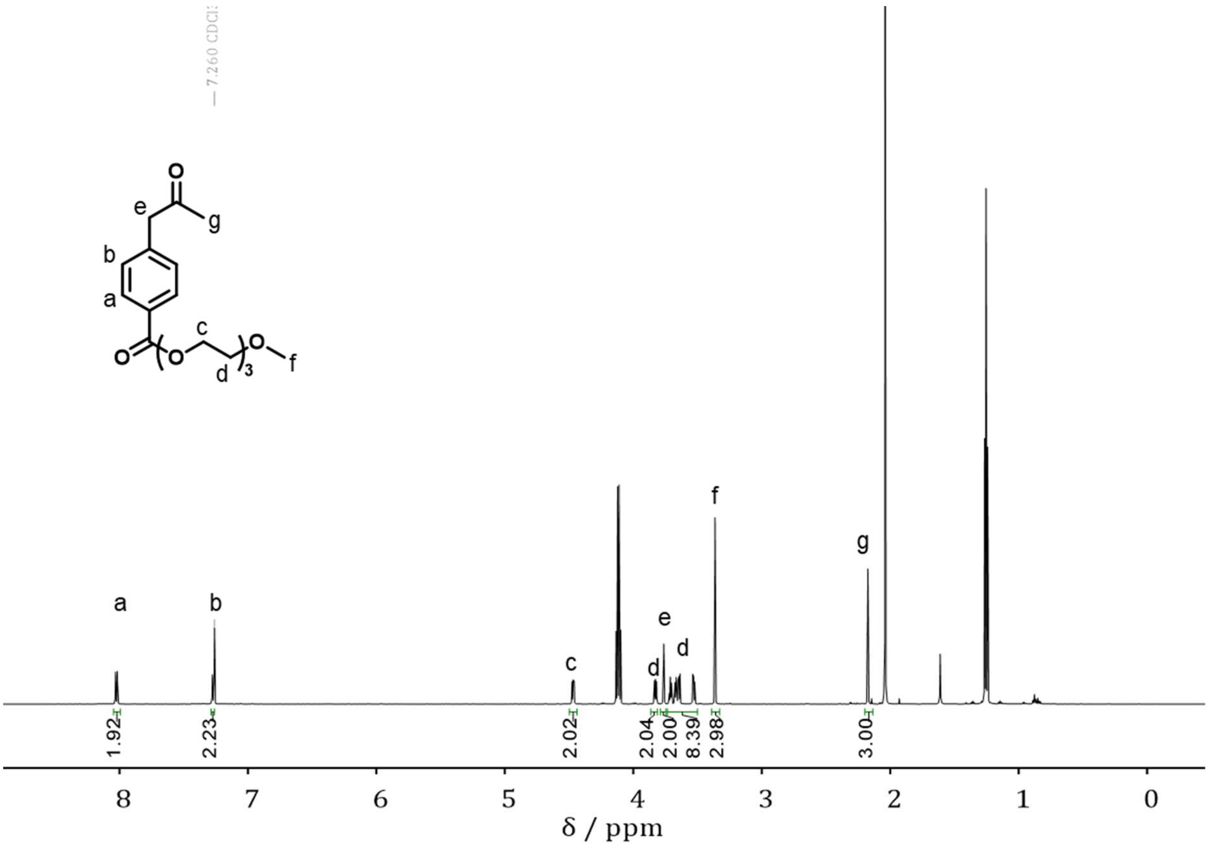

Figure 25 ^{1}H NMR spectrum of 2-(2-(2-methoxyethoxy)ethoxy)ethyl 4-(2-oxopropyl)benzoate recorded in $CDCl_3$ and assigned resonances, the solution contains a small amount of EtOAc.

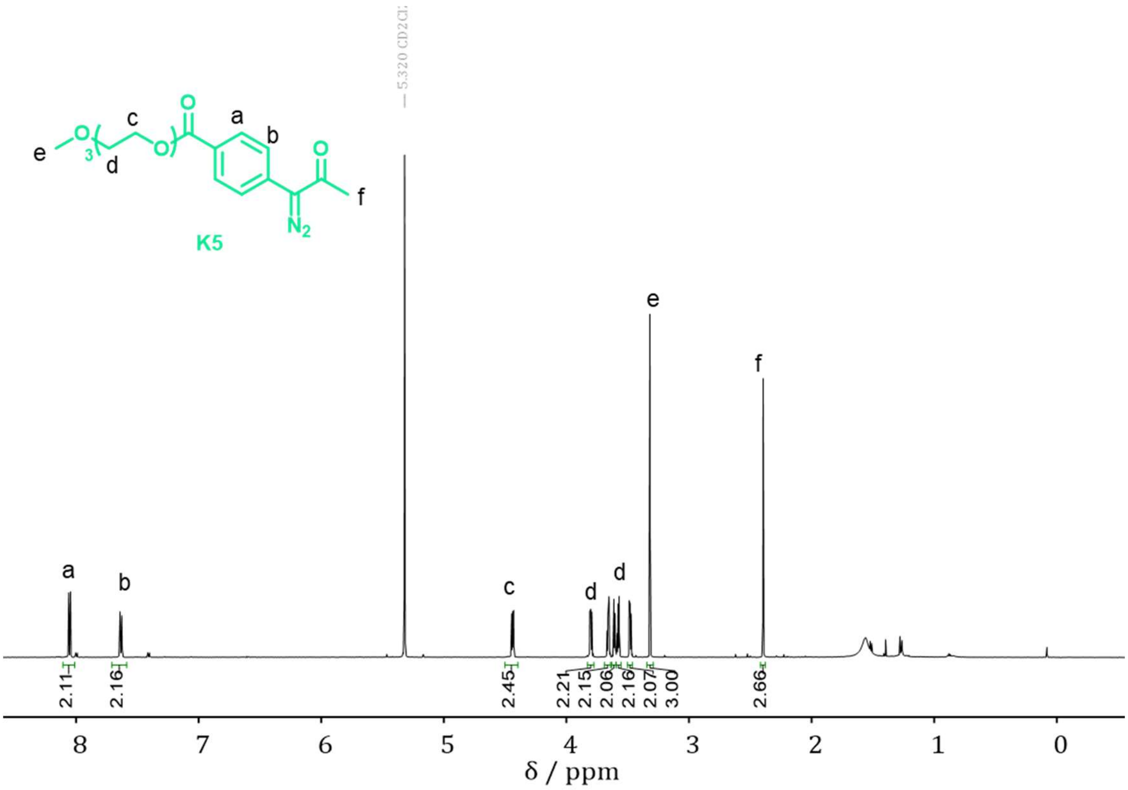

Figure 26 ^{1}H NMR spectrum of **K5** recorded in $CDCl_3$ and assigned resonances.

2.9 Photoproducts

1,2-bis(2,6-dichlorophenyl)-4-methyl-4-phenyl-1,2-diazetidin-3-one **P1**

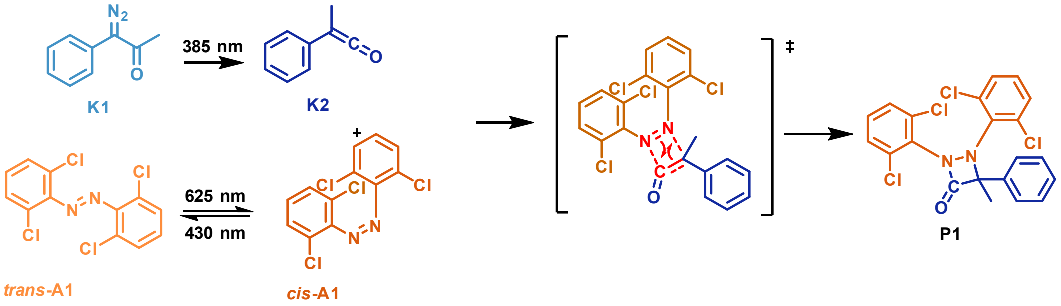

The synthesis of **P1** was conducted in a batch setup using a 1.5 mL crimp vial. The ketene (**K1**, 5.0 mg, 32 µmol, 2.0 eq) and the azobenzene (***trans*-A1**, 5.0 mg, 16 µmol, 1.0 eq) were dissolved in dichloromethane (1.5 mL, 15 mmol/L) and irradiated for 30 min simultaneously with 385 nm (1 A, 20 V, 2 cm distance) from one side 625 nm (2.1 A, 22 V, 2 cm distance) from the other side. The LEDs were cooled using a stream of air and a fan. After completion the solvent was removed under reduced pressure and submitted to reverse phase HPLC

(Acetonitrile:Water gradient from 5:95 to 100:0). The product was obtained as a yellow/orange oil (5.6 mg, 79% yield).

^{1}H NMR (600 MHz, Methylene Chloride-d_2) δ 7.55 – 7.44 (m, 2H), 7.35 (s, 2H), 7.29 (dt, J = 4.3, 1.8 Hz, 4H), 7.13 (t, J = 8.1 Hz, 1H), 6.92 (t, J = 7.8 Hz, 2H), 2.17 (s, 3H).

^{13}C NMR (151 MHz, Methylene Chloride-d_2) δ 165.82, 135.85, 130.67, 130.08, 129.23, 128.79, 128.59, 127.76, 89.48, 27.48.

ESI-HRMS (m/z): calculated for $[C_{21}H_{14}Cl_4N_2O+H]^+$: 450.9933, found 450.9930.

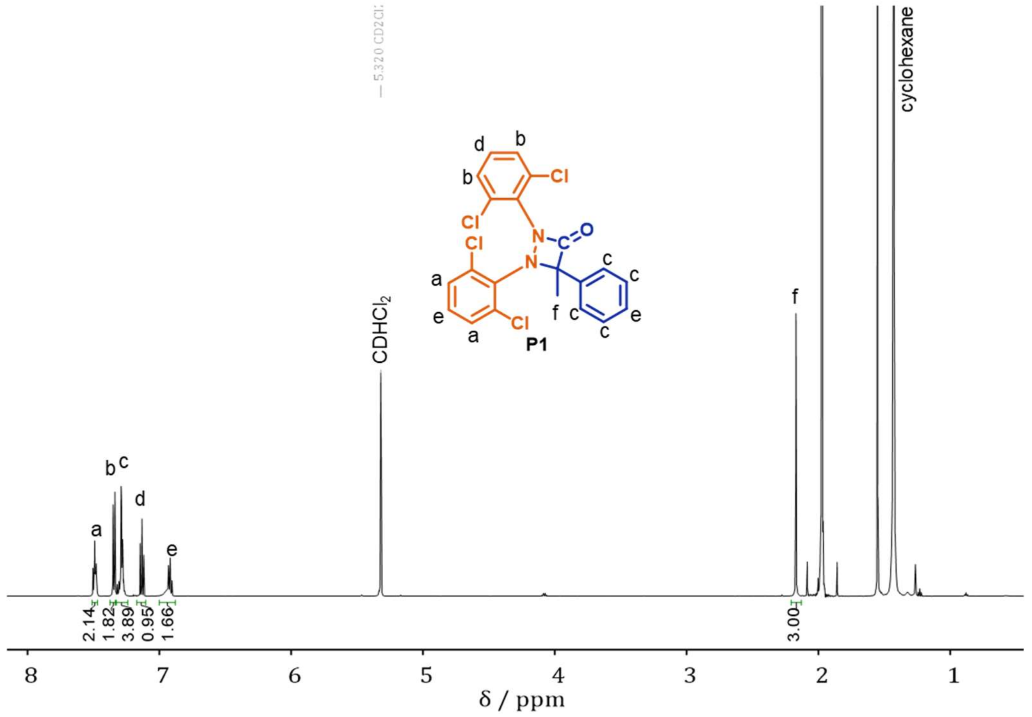

Figure 27 ^{1}H NMR spectrum of **P1** recorded in CD_2Cl_2 and assigned resonances.

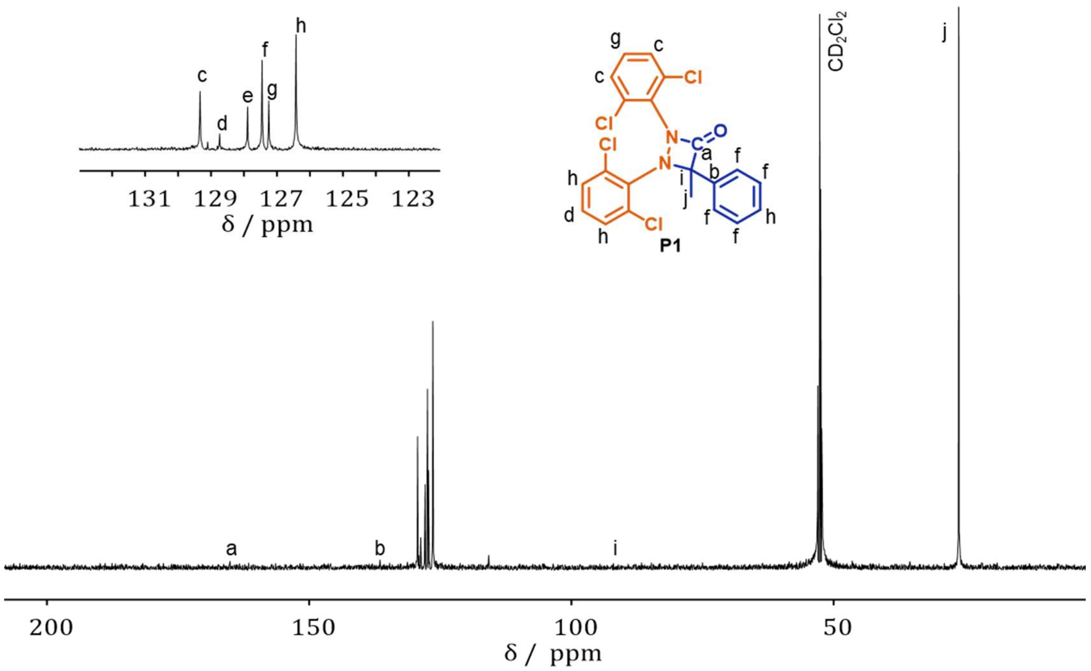

Figure 28 ^{13}C NMR spectrum of **P1** recorded in CD_2Cl_2 and assigned resonances.

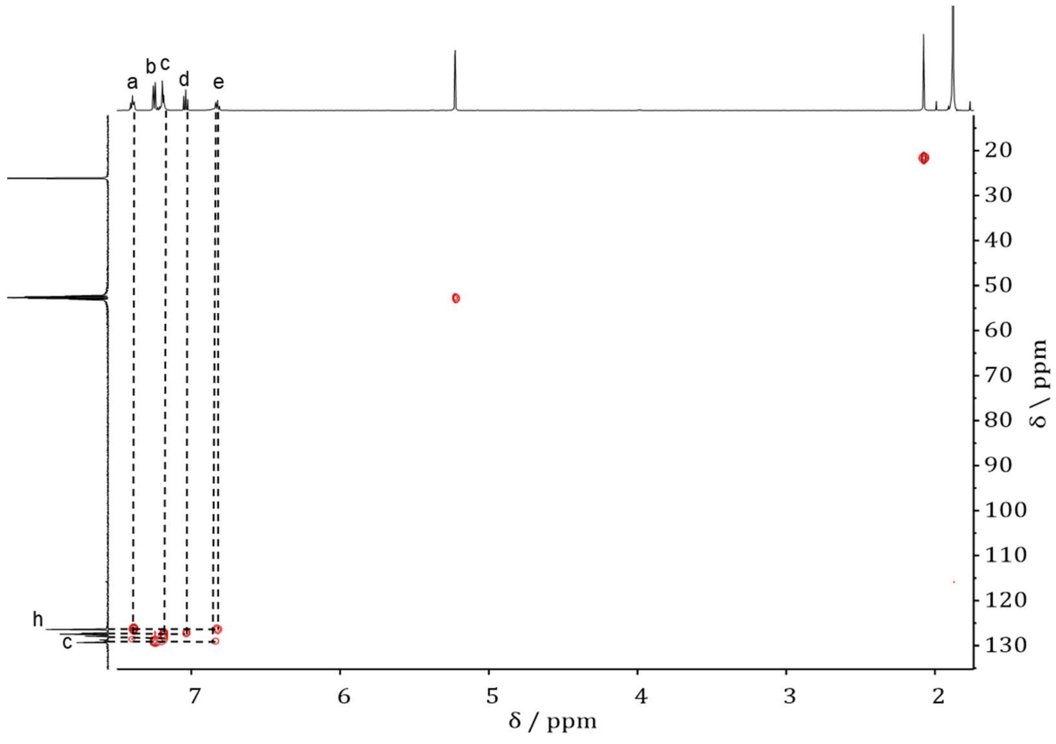

Figure 29 ^{1}H/^{13}C HSQC spectrum of **P1** recorded in CD_2Cl_2. The relevant cross resonances are assigned.

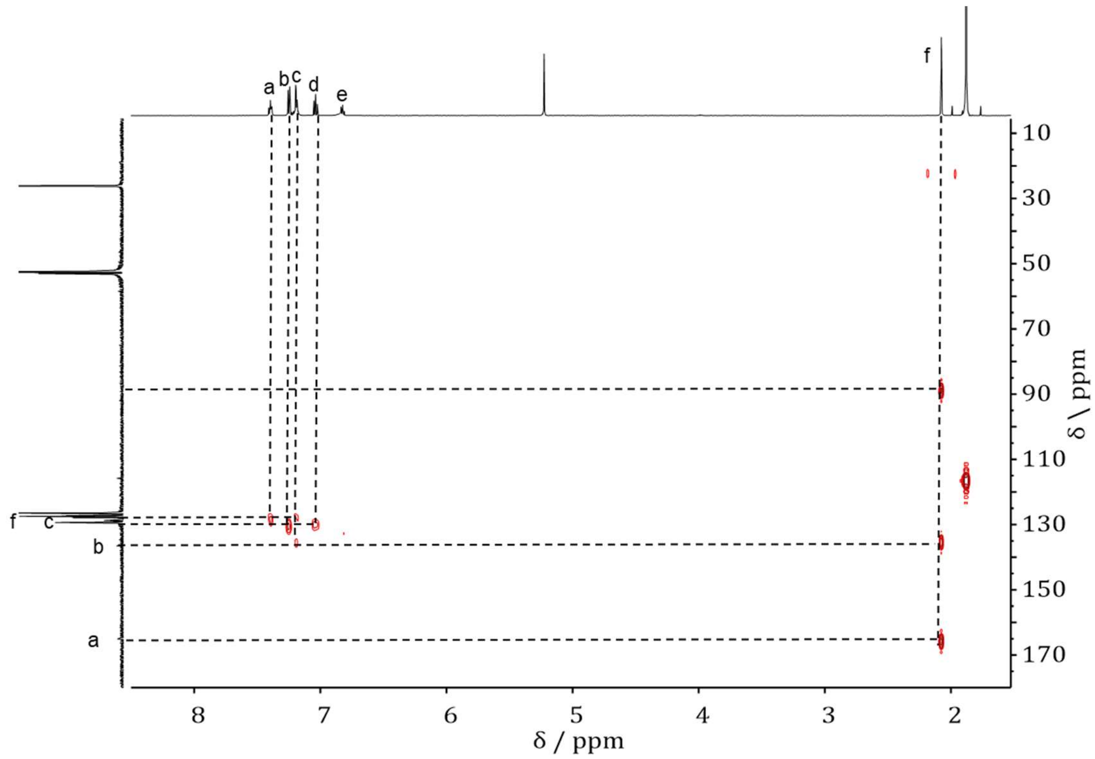

Figure 30 $^1H/^{13}C$ HMBC spectrum of **P1** recorded in CD_2Cl_2. The relevant cross resonances are assigned.

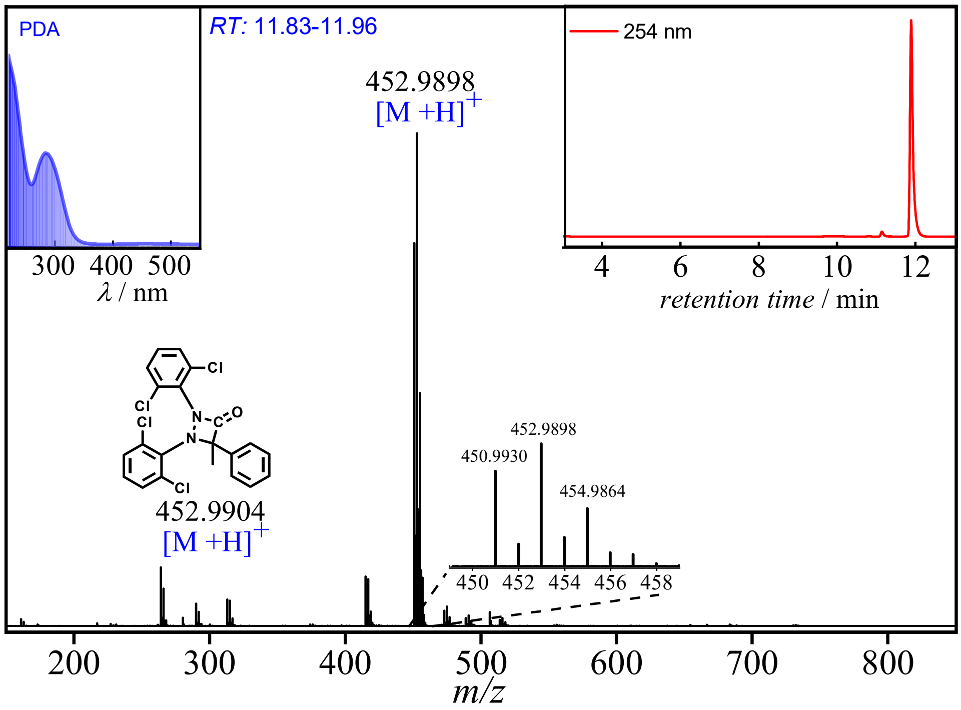

Figure 31 LC trace (254 nm detector wavelength), accumulated mass spectra and accumulated PDA spectra of **P1**.

methyl 3,5-dichloro-4-(2-(2,6-dichlorophenyl)-4-methyl-3-oxo-4-phenyl-1,2-diazetidin-1-yl) benzoate **P2**

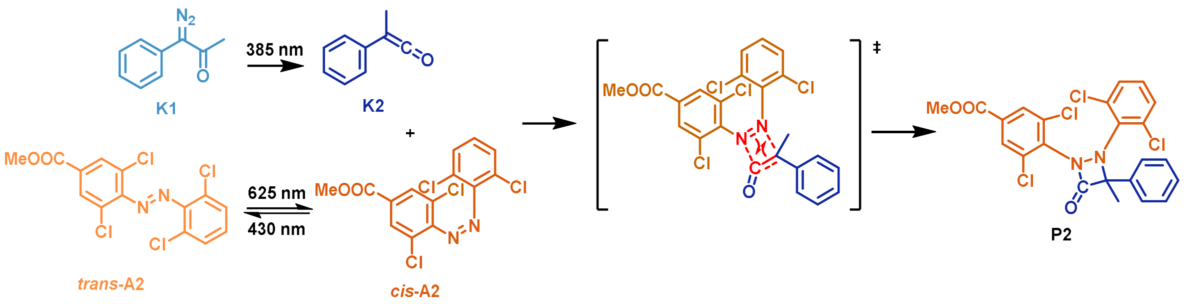

The synthesis of **P2** was conducted in a batch setup using a 1.5 mL crimp vial. The ketene (**K1**, 3.0 mg, 22 µmol, 2.0 eq) and the azobenzene (***trans*-A2**, 4.3 mg, 11 µmol, 1.0 eq) were dissolved in dichloromethane (1.5 mL, 15 mmol/L) and irradiated for 30 min simultaneously with 385 nm (1 A, 20 V, 2 cm distance) from one side 625 nm (2.1 A, 22 V, 2 cm distance) from the other side. The LEDs were cooled using a stream of air and a fan. After completion the solvent was removed under reduced pressure and submitted to reverse phase HPLC (Acetonitrile:Water gradient from 5:95 to 100:0). The product was obtained as a yellow/orange oil (3.9 mg, 67% yield).

^{1}H NMR (600 MHz, Methylene Chloride-d_2) δ 7.96 (s, 2H), 7.54 – 7.45 (m, 4H), 7.35 (d, J = 8.1 Hz, 2H), 7.32 – 7.27 (m, 8H), 7.15 (t, J = 8.1 Hz, 2H), 6.93 (d, J = 7.8 Hz, 2H), 3.88 (s, 3H), 3.82 (s, 3H), 2.21 (s, 3H), 2.17 (s, 3H).

^{13}C NMR (151 MHz, Methylene Chloride-d_2) δ 168.70, 166.95, 137.86, 130.66, 130.19, 129.01, 128.68, 128.29, 128.16, 126.77, 52.38, 24.94.

ESI-HRMS (m/z): calculated for $[C_{23}H_{16}Cl_4N_2O_3+H]^+$: 508.9988, found 508.9987.

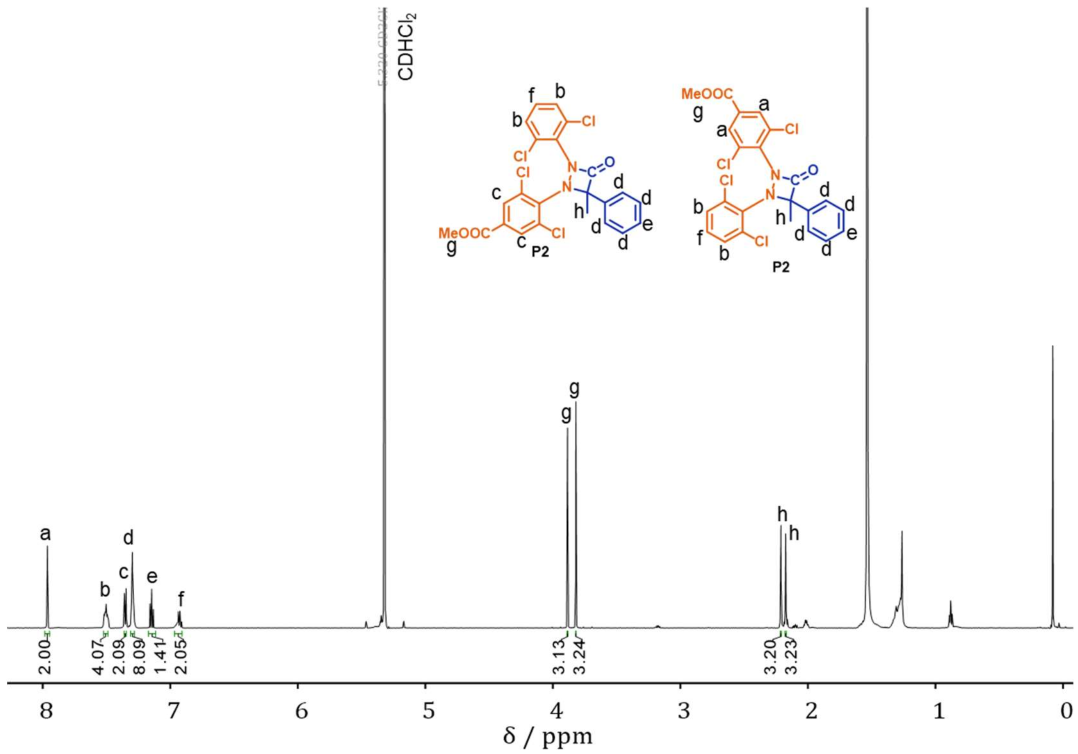

Figure 32 ^{1}H NMR spectrum of **P2** recorded in CD_2Cl_2 and assigned resonances.

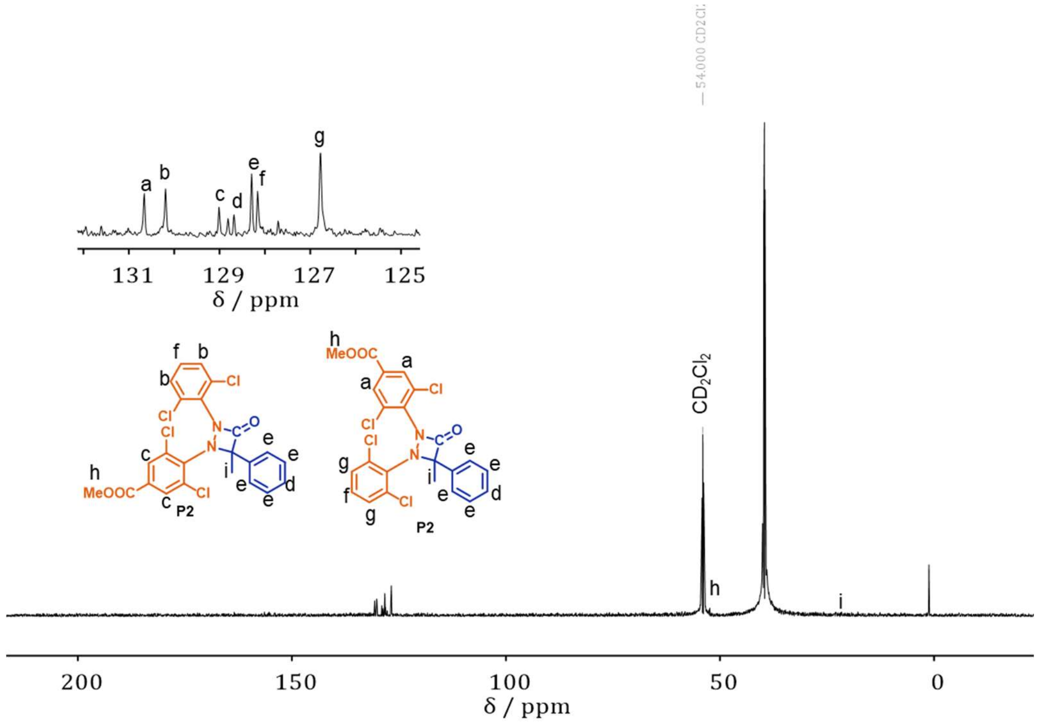

Figure 33 ^{13}C NMR spectrum of **P2** recorded in CD_2Cl_2 and assigned resonances.

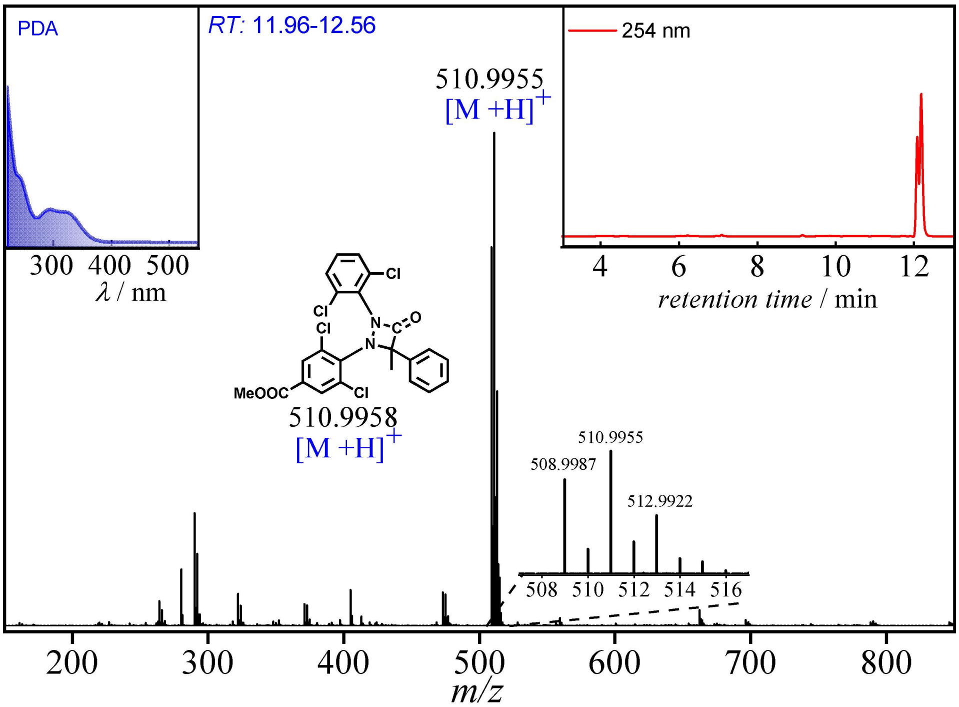

Figure 34 LC trace (254 nm detector wavelength), accumulated mass spectra and accumulated PDA spectra of **P2**.

methyl 3,5-dichloro-4-(2-(2,6-dichlorophenyl)-4-(4-methoxyphenyl)-3,6-dioxo-5-(trimethylsilyl)-3,6-dihydropyridazin-1(2H)-yl)benzoate **P4**

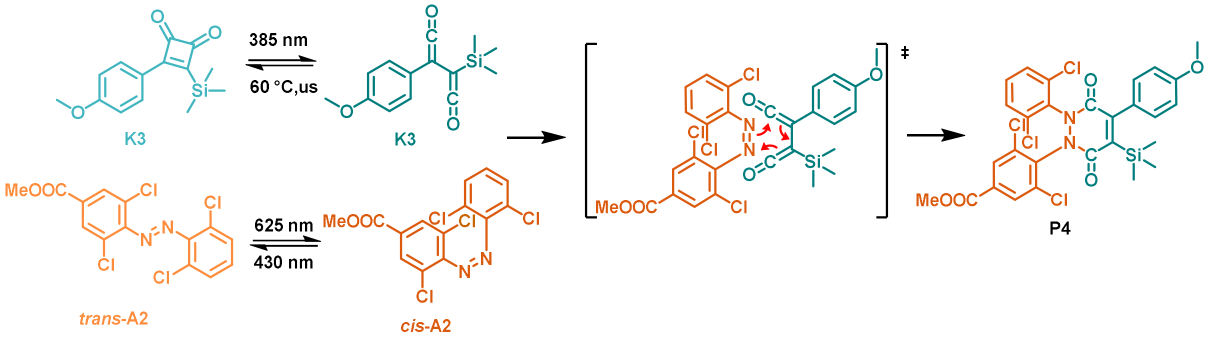

The synthesis of **P4** was conducted in a batch setup using a 1.5 mL crimp vial. The ketene (**K3**, 2.5 mg, 7.5 µmol, 3.0 eq) and the azobenzene (*trans*-**A2**, 5.1 mg, 18 µmol, 1.0 eq) were dissolved in dry chloroform (0.3 mL, 25 mmol/L) and irradiated for 10 min simultaneously with 385 nm (1 A, 20 V, 2 cm distance) from one side 625 nm (2.1 A, 22 V, 2 cm distance) from the other side. The LEDs were cooled using a stream of air and a fan. After completion the solvent was removed under reduced pressure and submitted to reverse phase HPLC

(Acetonitrile:Water gradient from 5:95 to 100:0). The product was obtained as a yellow oil (3.1 mg, 74% yield).

^{1}H NMR (600 MHz, Acetonitrile-d_3) δ 7.96 (d, J = 0.7 Hz, 2H), 7.41 (d, J = 1.9 Hz, 3H), 7.39 – 7.32 (m, 2H), 7.09 – 6.97 (m, 2H), 3.89 (d, J = 1.1 Hz, 3H), 3.84 (d, J = 0.8 Hz, 3H), -0.00 (d, J = 1.7 Hz, 9H).

^{13}C NMR (151 MHz, Acetonitrile-d_3) δ 163.33, 161.02, 152.68, 137.40, 134.29, 133.18, 132.58, 130.43, 130.09, 114.29, 56.06, 53.63, -0.04.

ESI-HRMS (m/z): calculated for $[C_{28}H_{24}Cl_4N_2O_5Si+H]^+$: 637.0281, found 637.0271

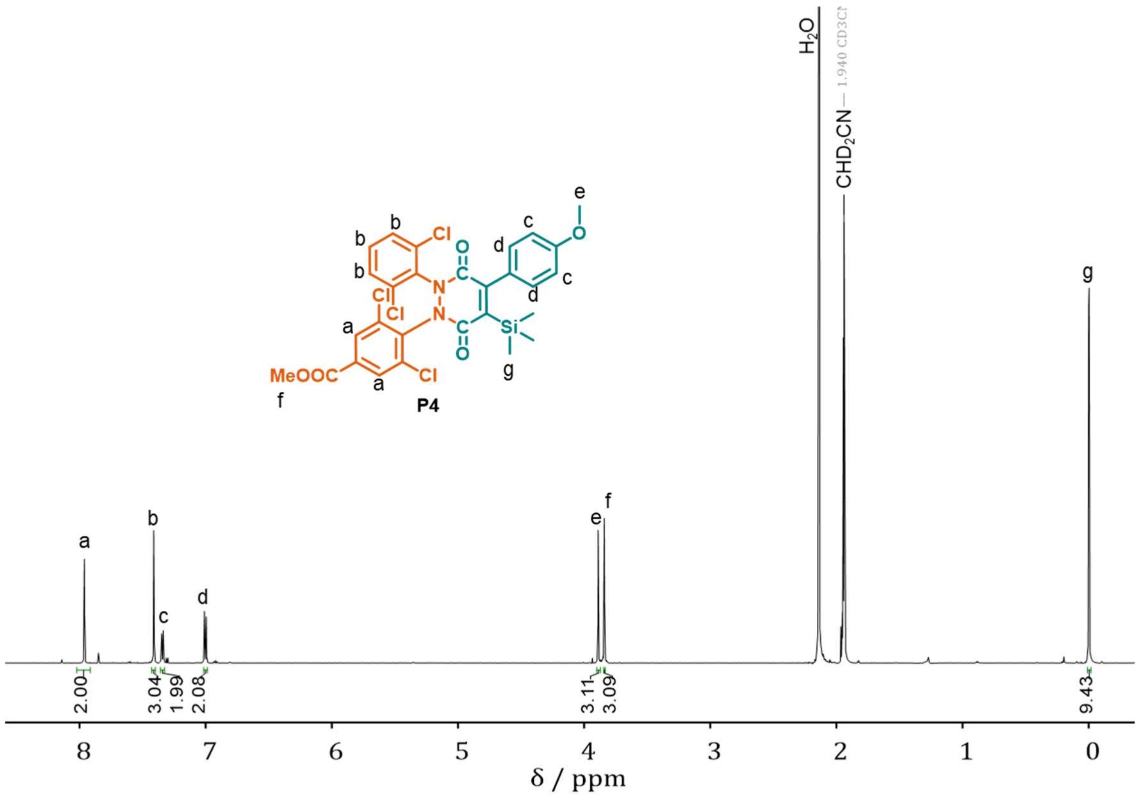

Figure 35 ^{1}H NMR spectrum of **P4** recorded in CD_3CN and assigned resonances.

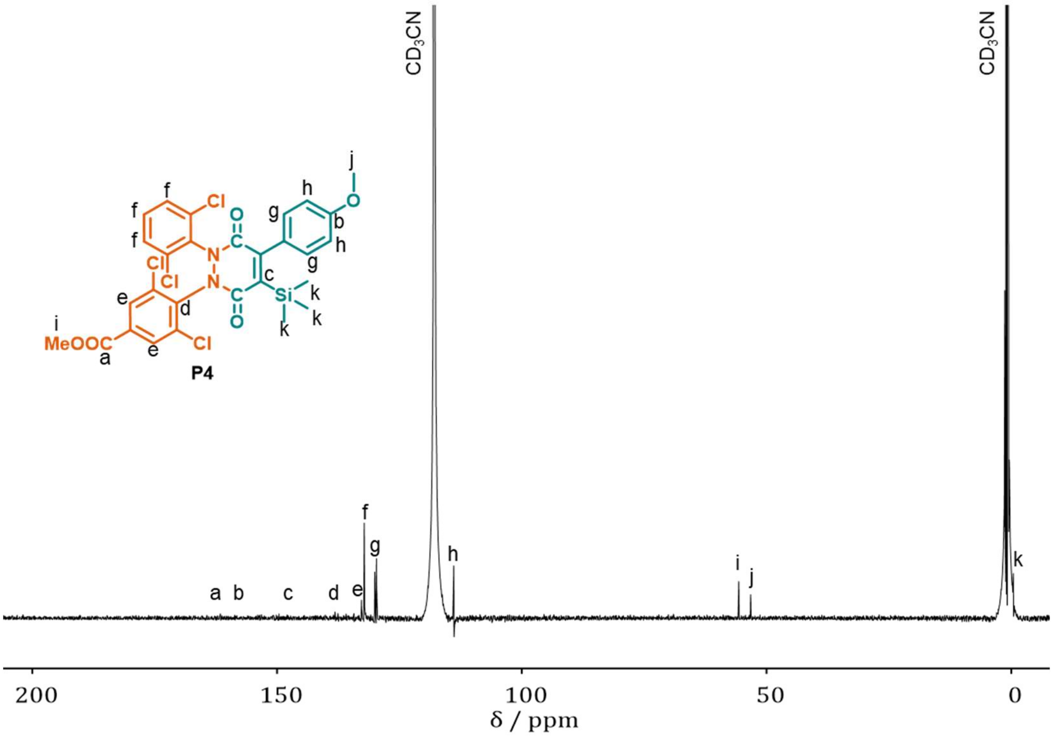

Figure 36 ^{13}C NMR spectrum of **P4** recorded in CD_3CN and assigned resonances.

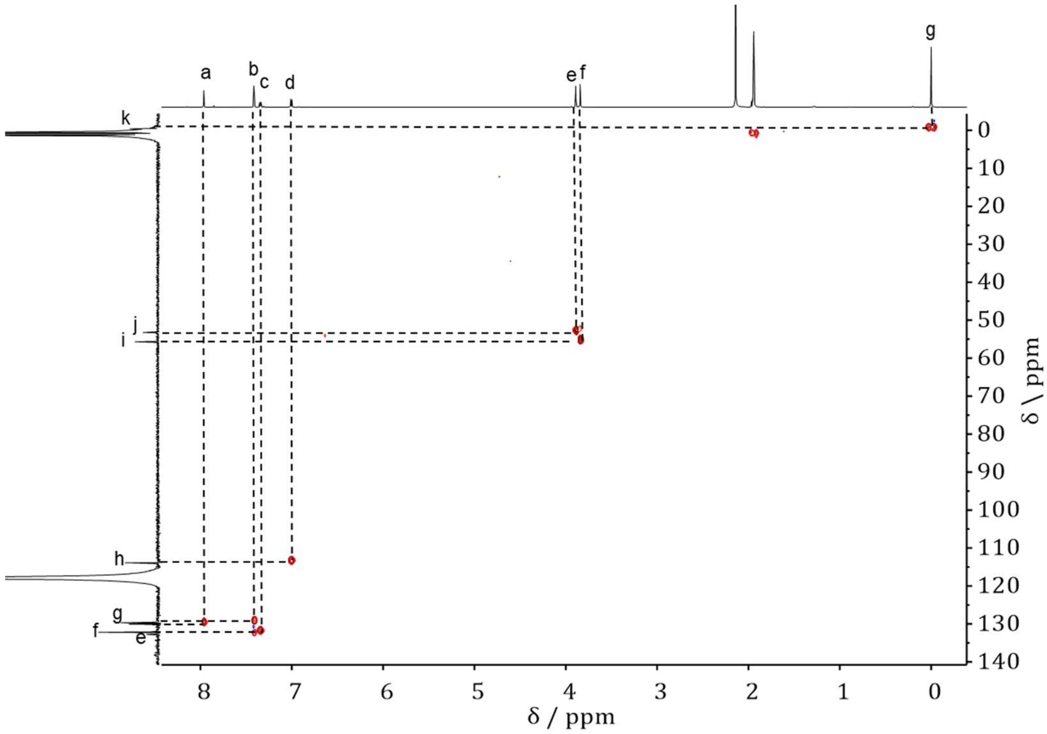

Figure 37 ^{1}H/^{13}C HSQC spectrum of **P4** recorded in CD_3CN. The relevant cross resonances are assigned.

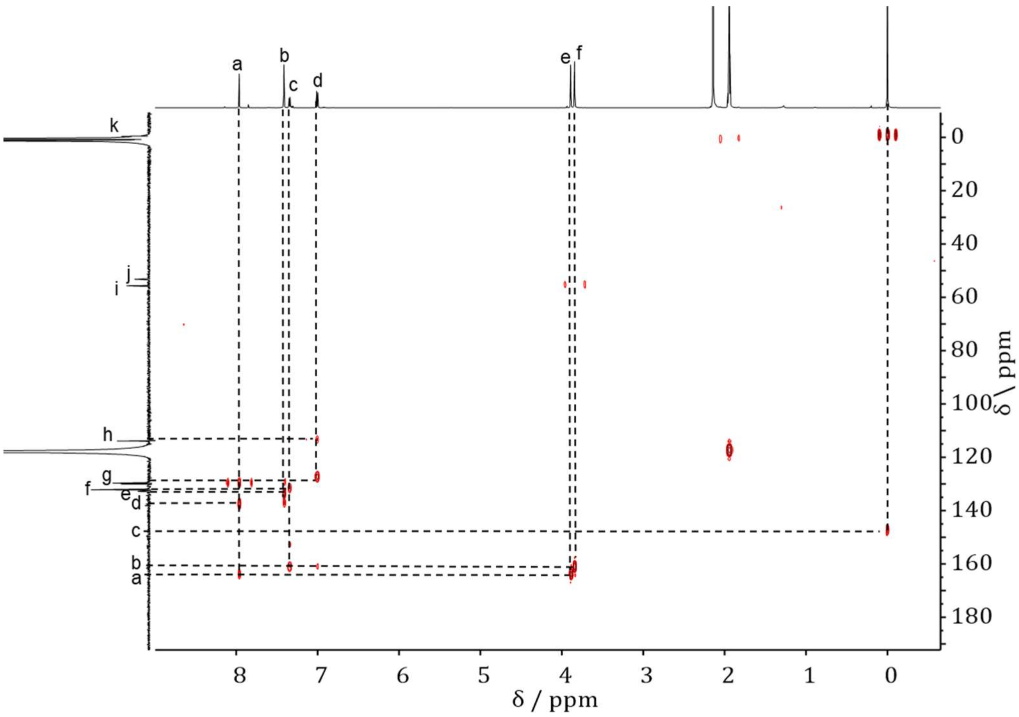

Figure 38 $^{1}H/^{13}C$ HMBC spectrum of **P4** recorded in CD_3CN. The relevant cross resonances are assigned.

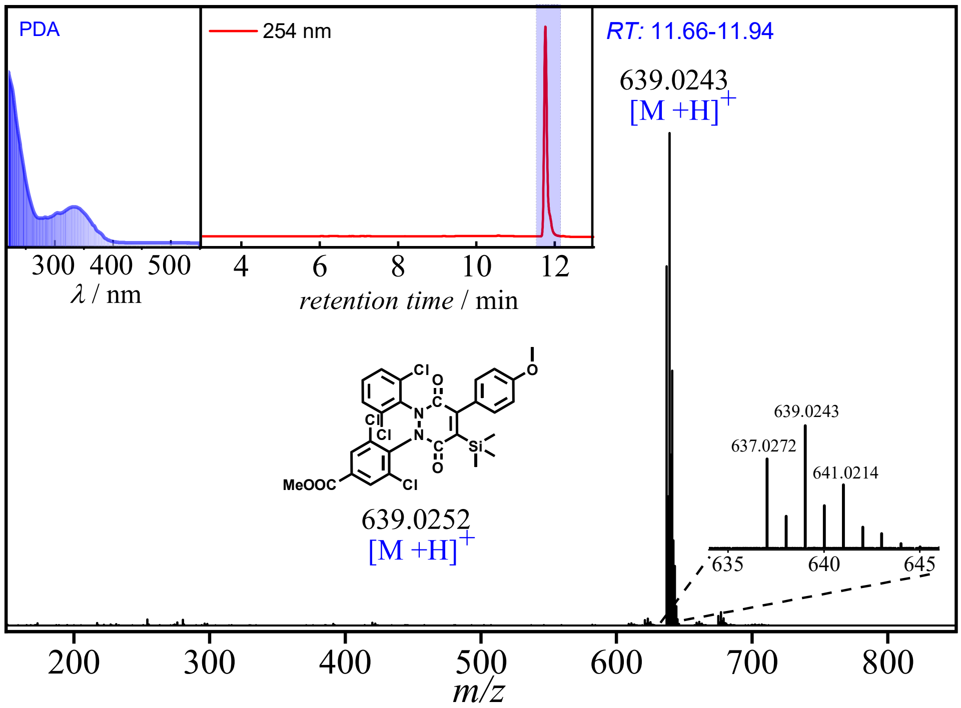

Figure 39 LC trace (254 nm detector wavelength), accumulated mass spectra and accumulated PDA spectra of **P4**.

3 Assessment of photochemical reactivity

3.1 Azobenzene

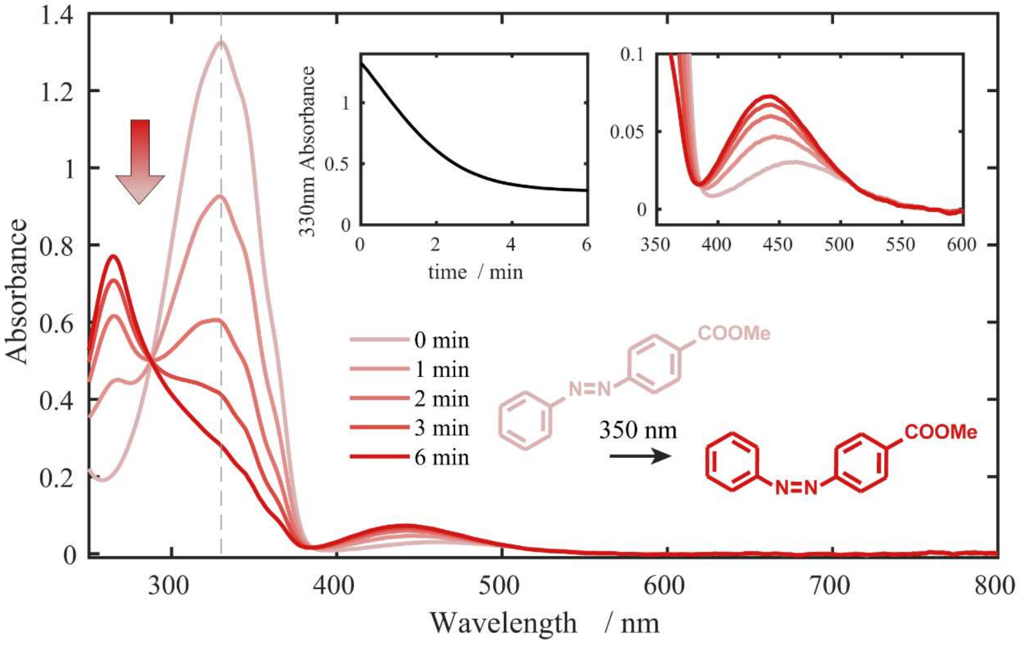

Figure 40 In-situ absorbance spectra of **A5** switching from the *trans* to *cis* isomer in chloroform during 350 nm irradiation (I = 17 mW). Left inset shows transient of 330 nm absorbance during irradiation. Right inset highlights low intensity region.

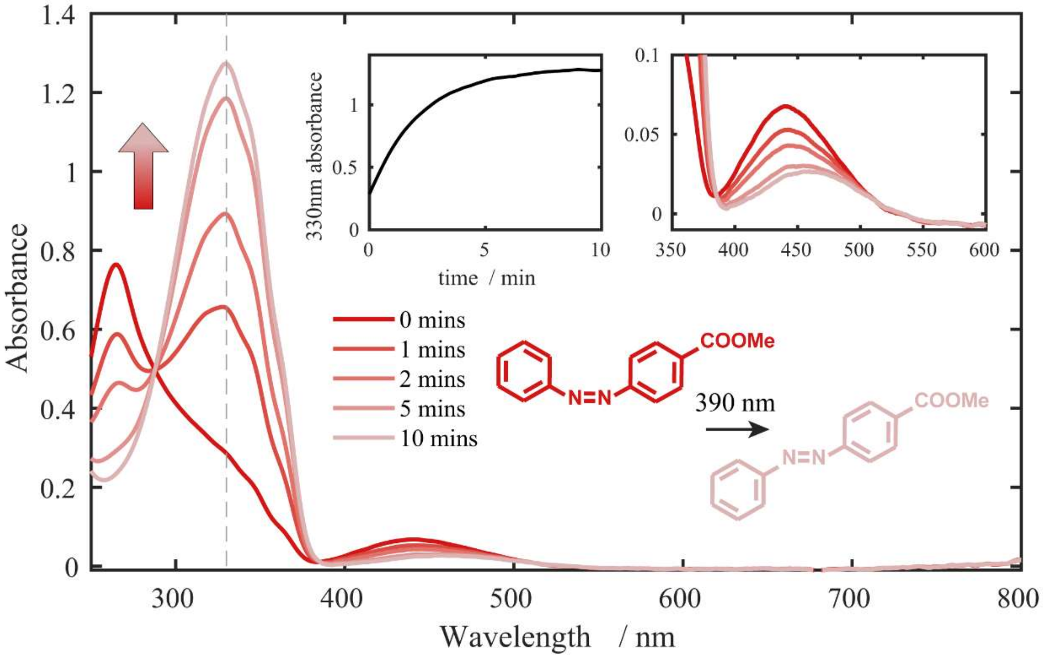

Figure 41 In-situ absorbance spectra of **A5** switching from *trans* to *cis* isomer in chloroform during 390 nm, 8.6 mW irradiation. Left inset shows transient of 330 nm absorbance during irradiation. Right inset highlights low intensity region.

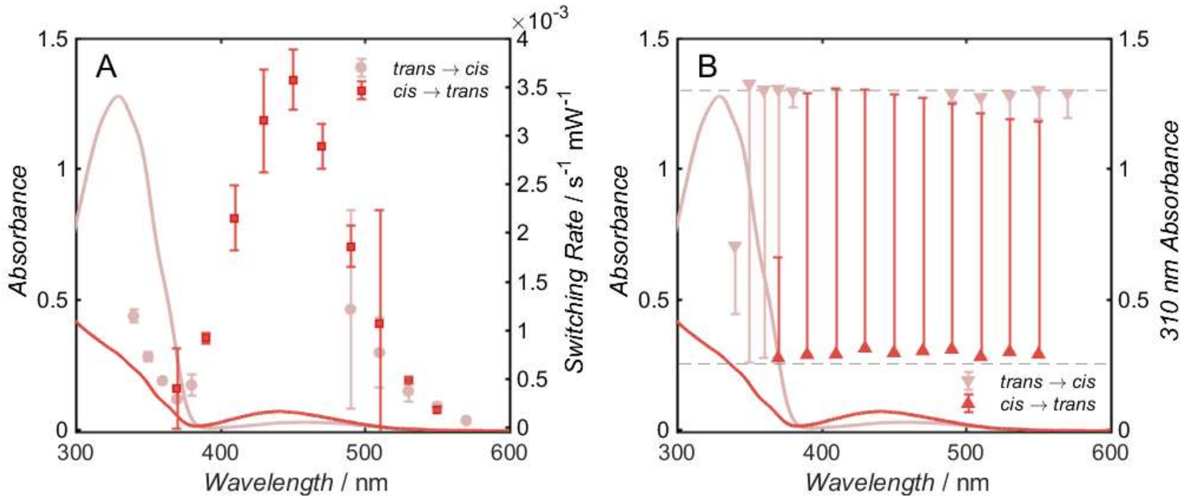

Figure 42 (A) wavelength dependent rates of photoisomerization of **A5,** normalised to the incident light intensity and overlayed with the absorbance spectra of *trans*-**A5** and *cis*-**A5.** Error bars indicate variation in least squares fit. (B) Wavelength dependent equilibrium in solution as determined from 330 nm absorbance signal. Triangle indicates intensity of 330 nm absorbance prior to irradiation and flat line indicates the final asymptotic 330 nm absorbance. A 330 nm absorbance of 1.3 (dashed, grey) is considered 100% *trans*-**A5** and a 330 nm absorbance of 0.3 (dashed, grey) is considered 100% *cis*-**A5**.

3.2 Cl-Azobenzene

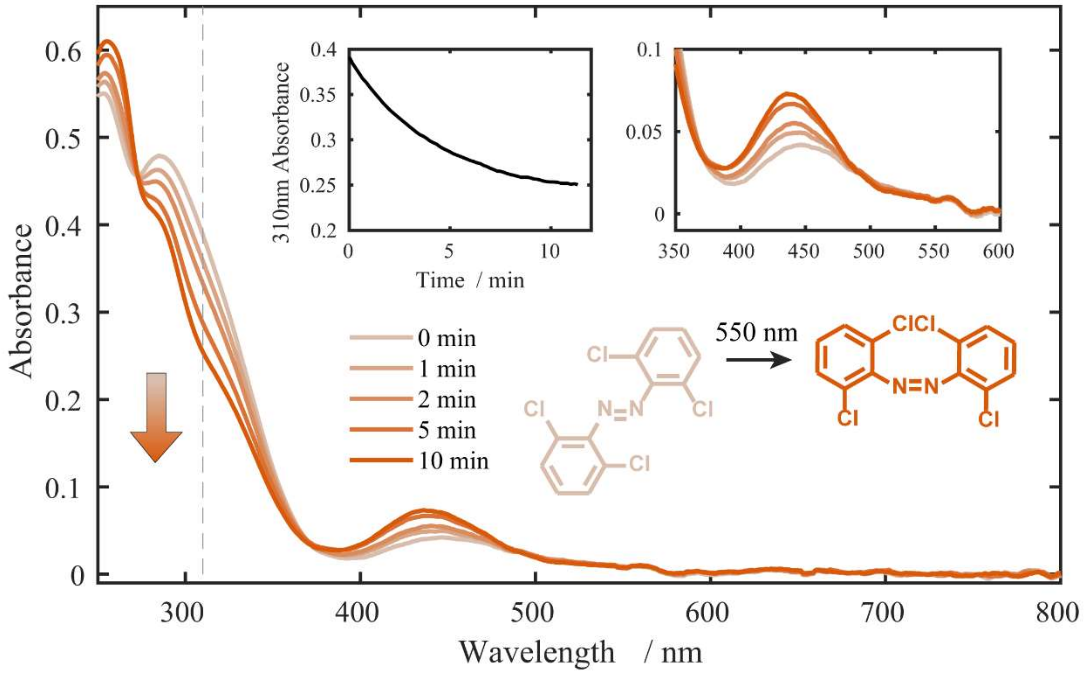

Figure 43 In-situ absorbance spectra of **A1** switching from the *trans* to *cis* isomer in DCM during 550 nm, 35 mW irradiation. Left inset shows transient of 310 nm absorbance during irradiation. Right inset highlights low intensity region.

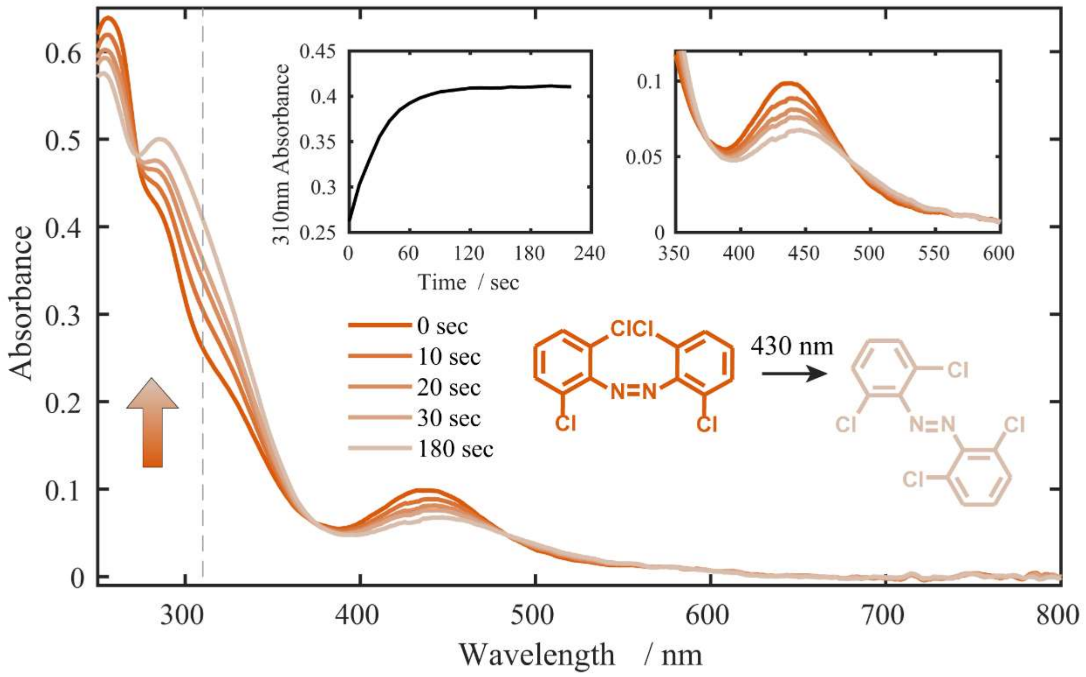

Figure 44 In-situ absorbance spectra of **A1** switching from the *cis* to *trans* isomer in DCM during 430 nm, 20 mW irradiation. Left inset shows transient of 310 nm absorbance during irradiation. Right inset highlights low intensity region.

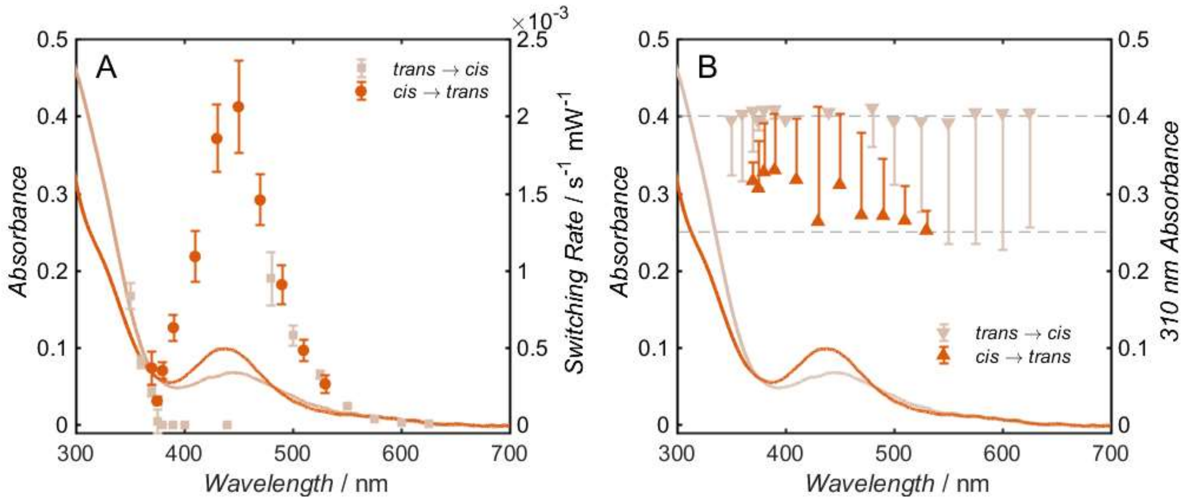

Figure 45 (A) wavelength dependent rates of photoisomerization of **A1**, normalised to the incident light intensity and overlayed with the absorbance spectra of *trans*-**A1** and *cis*-**A1**. Error bars indicate variation in least squares fit. (B) Wavelength dependent equilibrium in solution as determined from 310 nm absorbance signal. Triangle indicates intensity of 310 nm absorbance prior to irradiation and flat line indicates the final asymptotic 310 nm absorbance. A 310 nm absorbance of 0.4 (dashed, grey) is considered 100% *trans*-**A1** and a 310 nm absorbance of 0.25 (dashed, grey) is considered 100% *cis*-**A1**.

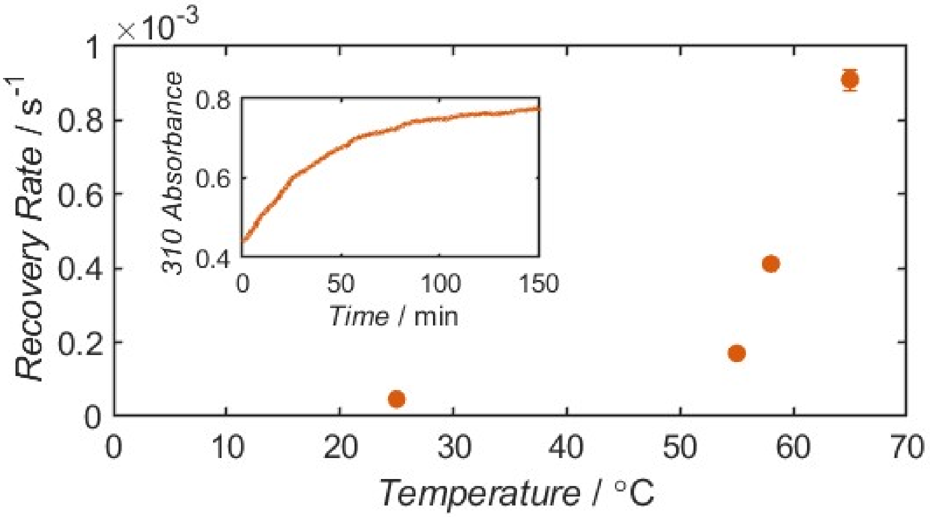

Figure 46 Temperature dependent rates of isomerisation of *cis*-**A1** to *trans*-**A1** in DCM. Error bars indicate variation in least squares fit. Inset shows kinetic of 310 nm absorbance during thermal recovery at 65 °C.

3.3 Photoactive Ketone

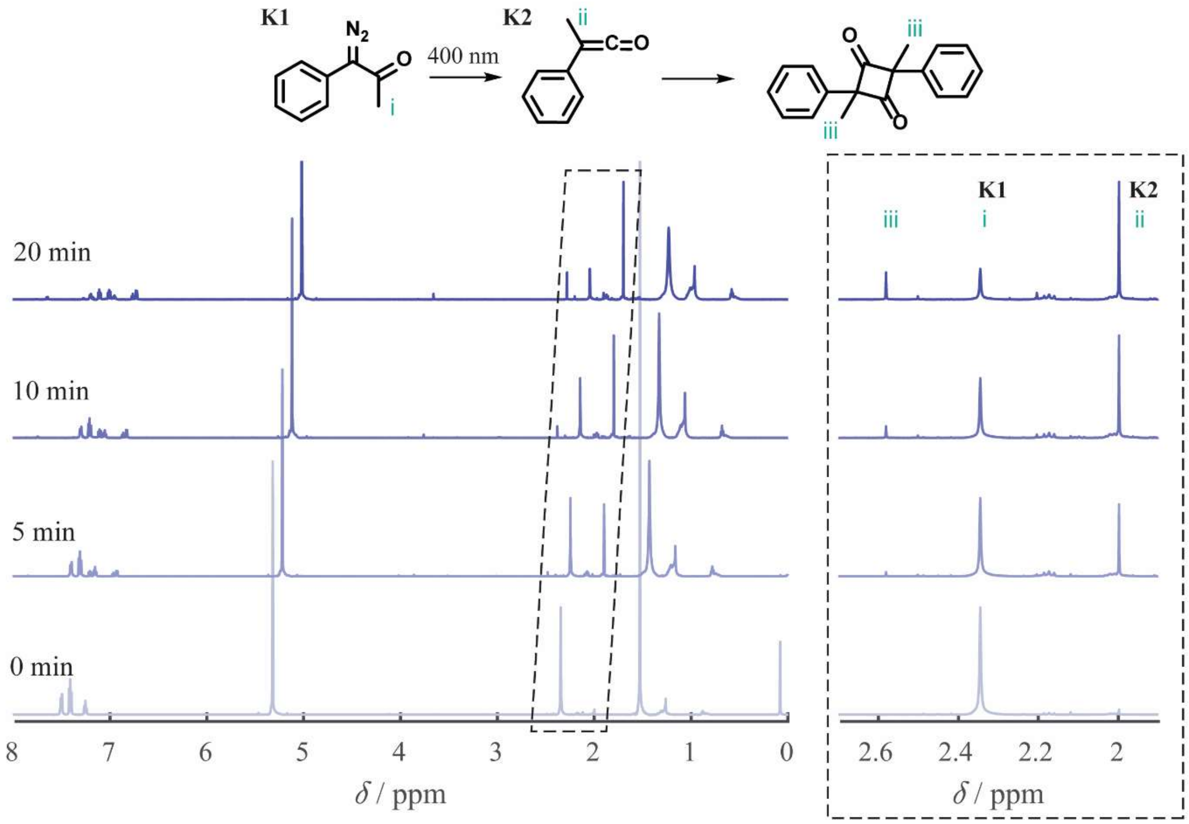

Figure 47 ^{1}H-NMR spectra tracking conversion of **K1** to **K2** over time during irradiation with 400 nm, 280 ± 20 μJ pulses from a 20 Hz tuneable OPO.

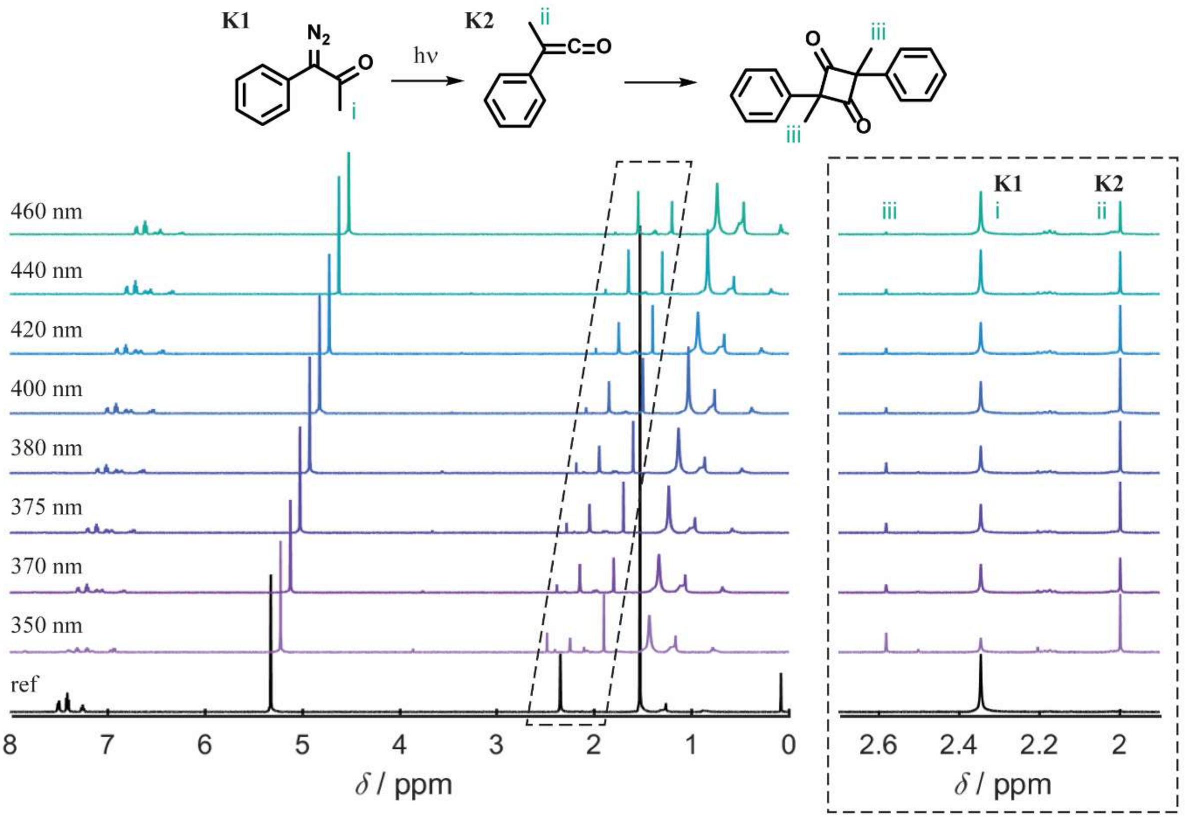

Figure 48^{1}H-NMR spectra tracking conversion of **K1** to **K2** after irradiation with (3.8 ± 0.3) x 10^{18} photons of various wavelengths from a 20 Hz tuneable OPO.

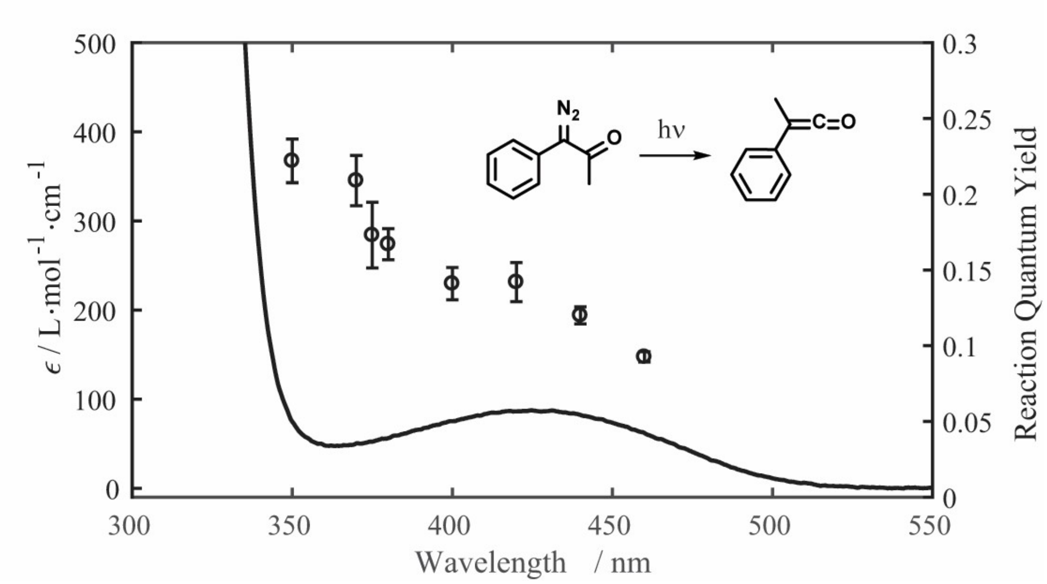

Figure 49 Wavelength dependent reaction quantum yield of photoactivated ketene formation. Conversions determined from ^{1}H-NMR spectra **K1** to **K2** after irradiation with (3.8 ± 0.3)x10^{18} photons of various wavelengths from a 20 Hz tuneable OPO. Error bars determined from standard deviation in pulse energy during irradiation.

3.4 Photoreversible ketene

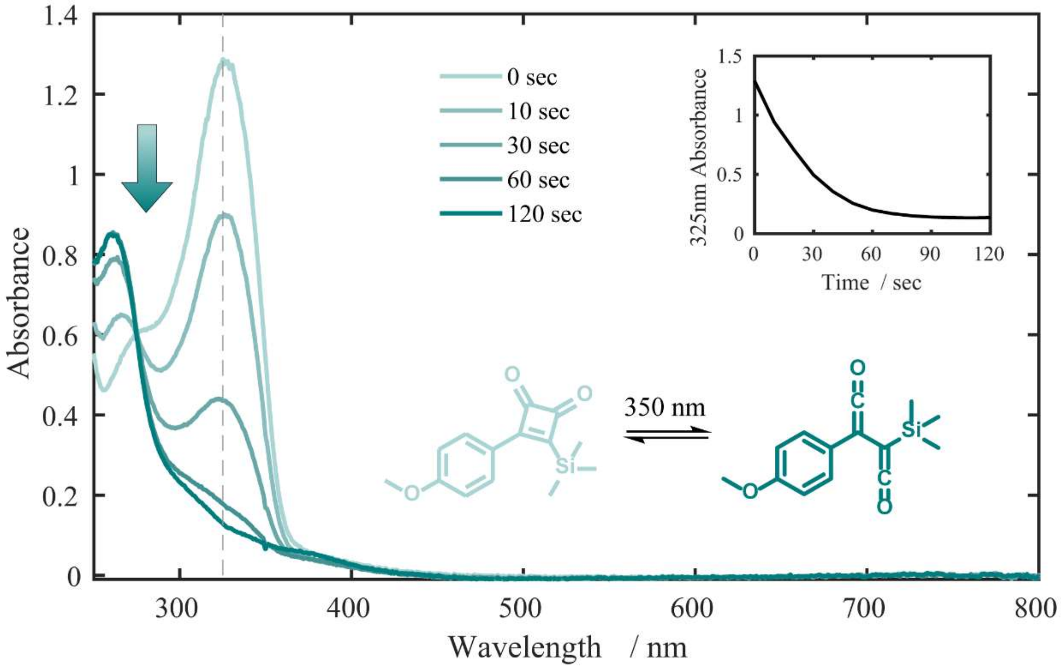

Figure 50 In-situ absorbance spectra during light activation of **K3** in chloroform during 350 nm, 16 mW irradiation. Inset shows transient of 325 nm absorbance during irradiation.

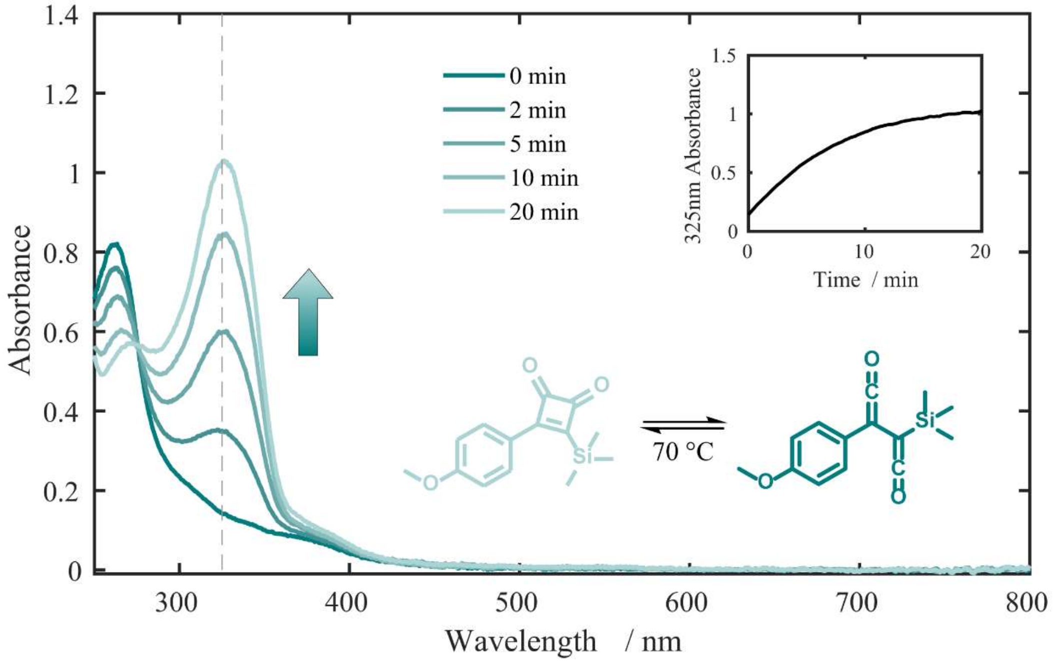

Figure 51 In-situ absorbance spectra during thermal recovery of **K3** at 70 °C. Inset shows transient of 325 nm absorbance during thermal recovery.

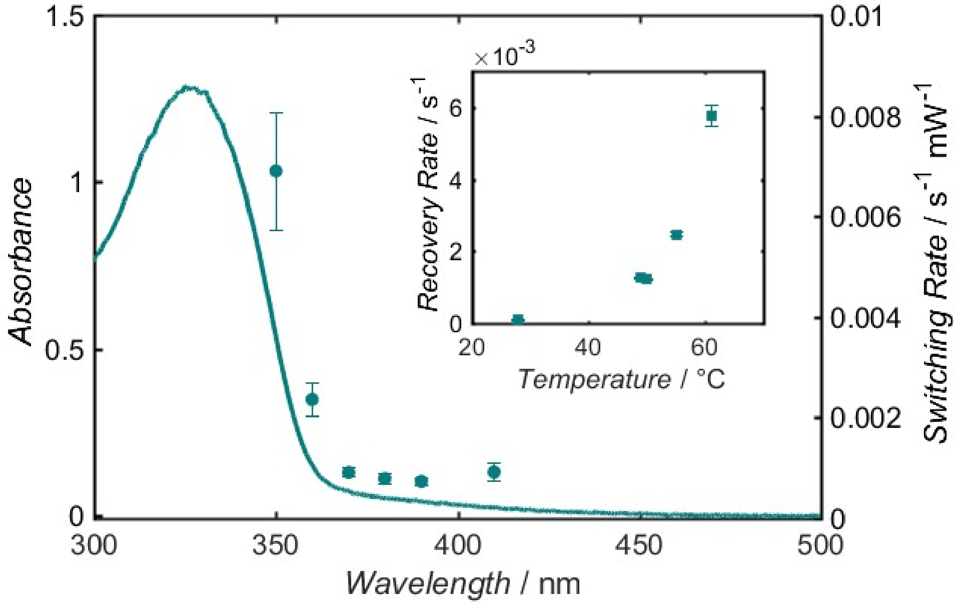

Figure 52 Wavelength dependent rates of photoactivation of **K3** in chloroform, normalised by the incident light intensity and overlayed with the absorbance spectra of **K3**. Inset shows the temperature dependent thermal recovery rate of **K3** in chloroform. Error bars indicate variation in least squares fit.

4 Polymer End Group Modification

For the polymer endgroup modification, poly(ethylene glycol) azobenzene **A3** (5.0 mg, 2.2 µmol, 1.0 eq) and ketene **K1** (2.0 mg, 11.4 µmol, 5.0 eq) were dissolved in deuterated DCM (0.6 mL) in a NMR tube. The solution was irradiated for 30 min simultaneously with 385 nm (1 A, 20 V, 2 cm distance) from one side 625 nm (2.1 A, 22 V, 2 cm distance) from the other side. For the control experiments, the same setup was used while only one wavelength was turned on, respectively. Upon completion, the solvent was removed under reduced pressure and the polymer was analysed by size-exclusion chromatography (SEC), size-exclusion chromatography mass spectrometry (SEC-MS) and NMR spectroscopy.

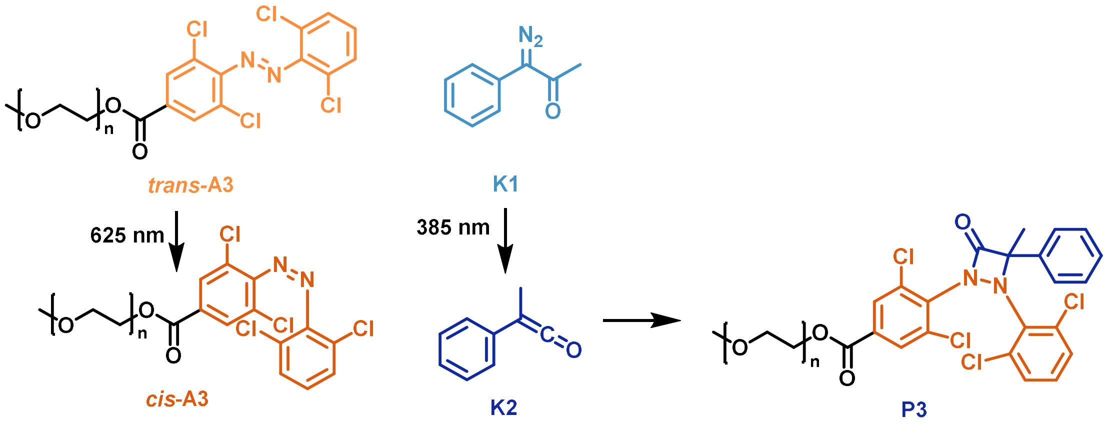

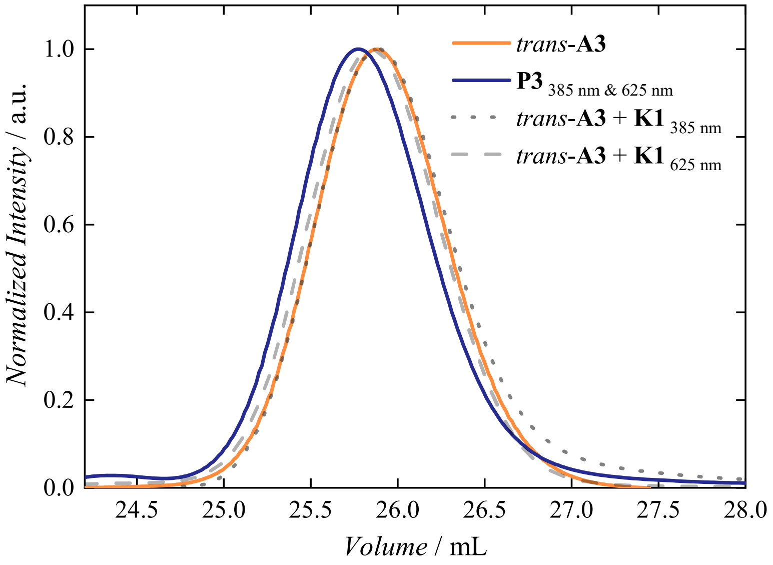

Figure 53 Size-exclusion chromatography of **P3** and after irradiation of **A3** and ketene (**K1**) with 385 and 625 nm. Control experiments were conducted using one wavelength, respectively.

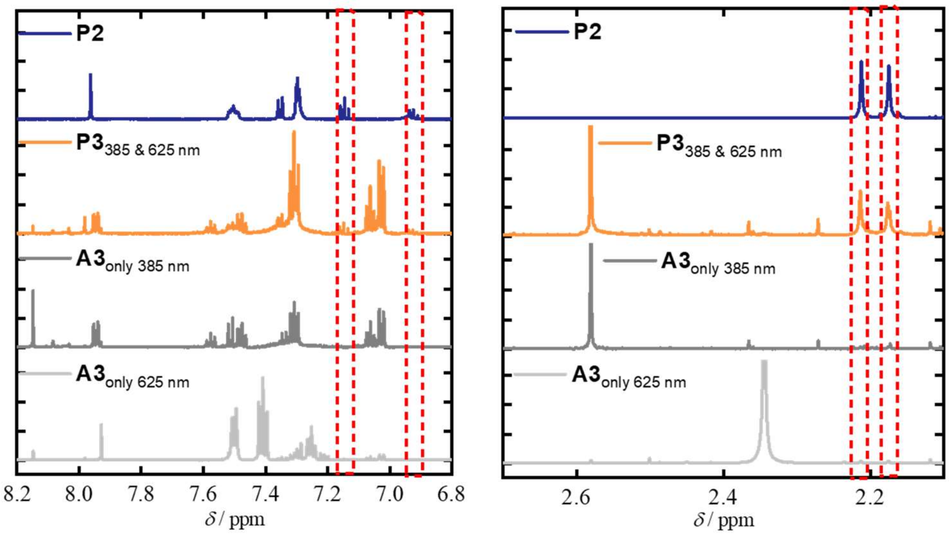

Figure 54 Comparison of the ^{1}H NMR spectra of the isolated small molecule ketene-azobenzene (**P2**) and the spectra of the irradiation product **P3** of **A3** and ketene (**K2**) with 385 and 625 nm. Control experiments were conducted using only one wavelength, respectively. The key resonances are highlighted.

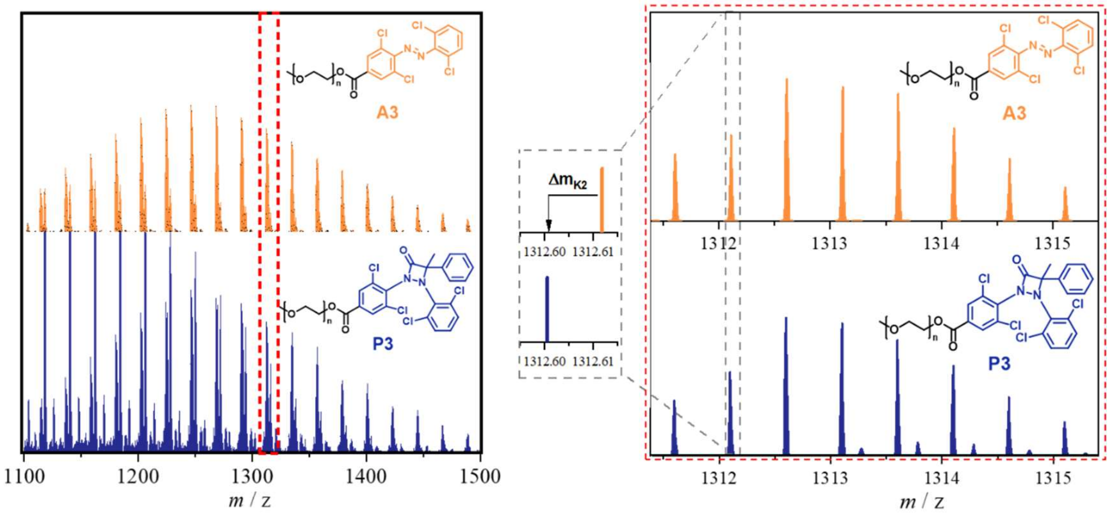

Figure 55 Comparison of the size-exclusion chromatography mass spectrometry (SEC-MS) spectra of **P3** with the spectra after the irradiation of **A3** and ketene (**K1**) with 385 and 625 nm. The right insert highlights the isotopic pattern and allows direct comparison of the weight gain upon endgroup modification.

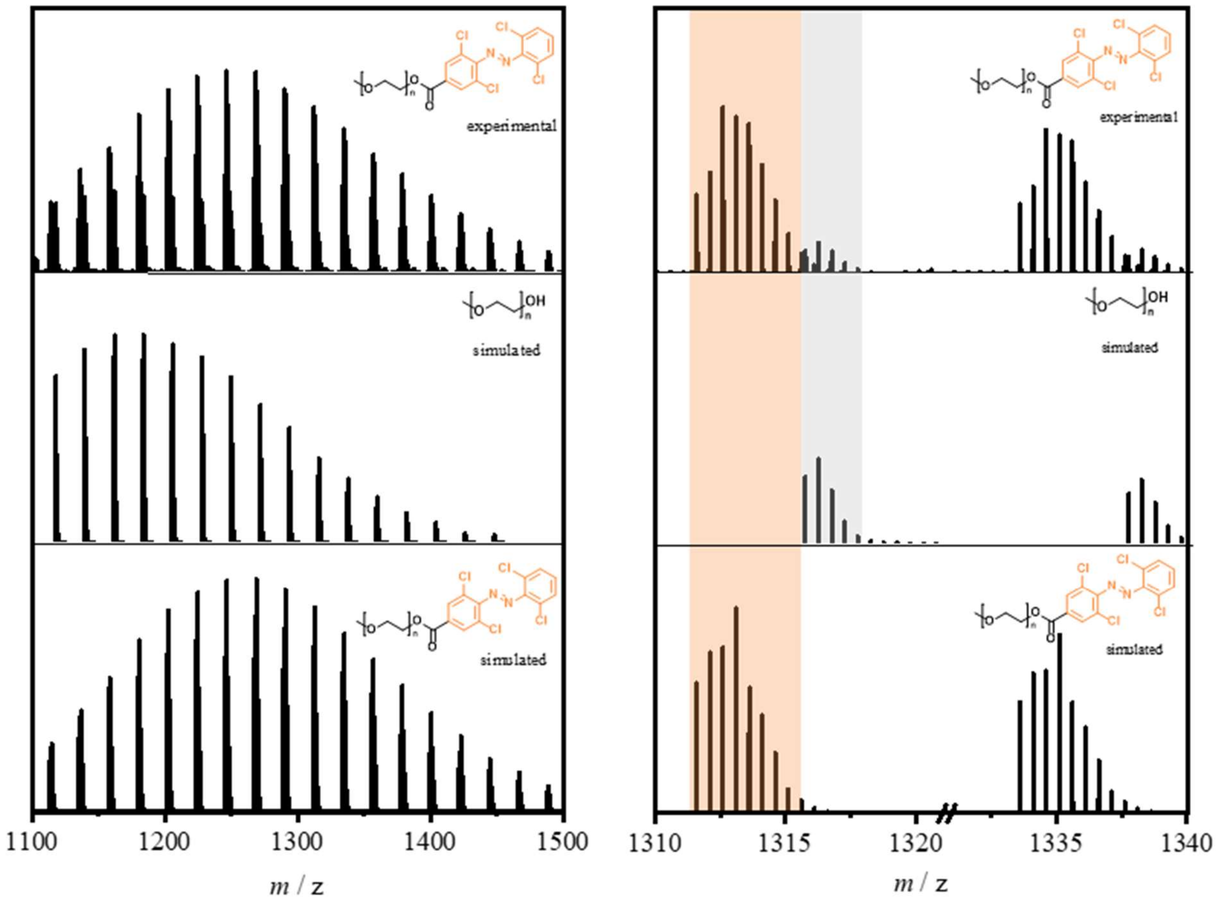

Figure 56 Comparison of the experimental SEC-MS spectra of **A3** with the simulated spectra of **A3** (orange). Additionally, the simulated spectrum of PEG-OH is depicted, matching the residual pattern (grey) in the experimental spectra of **A3**. The right zoom-in highlights a representative area and allows for precise comparison of the isotopic pattern.

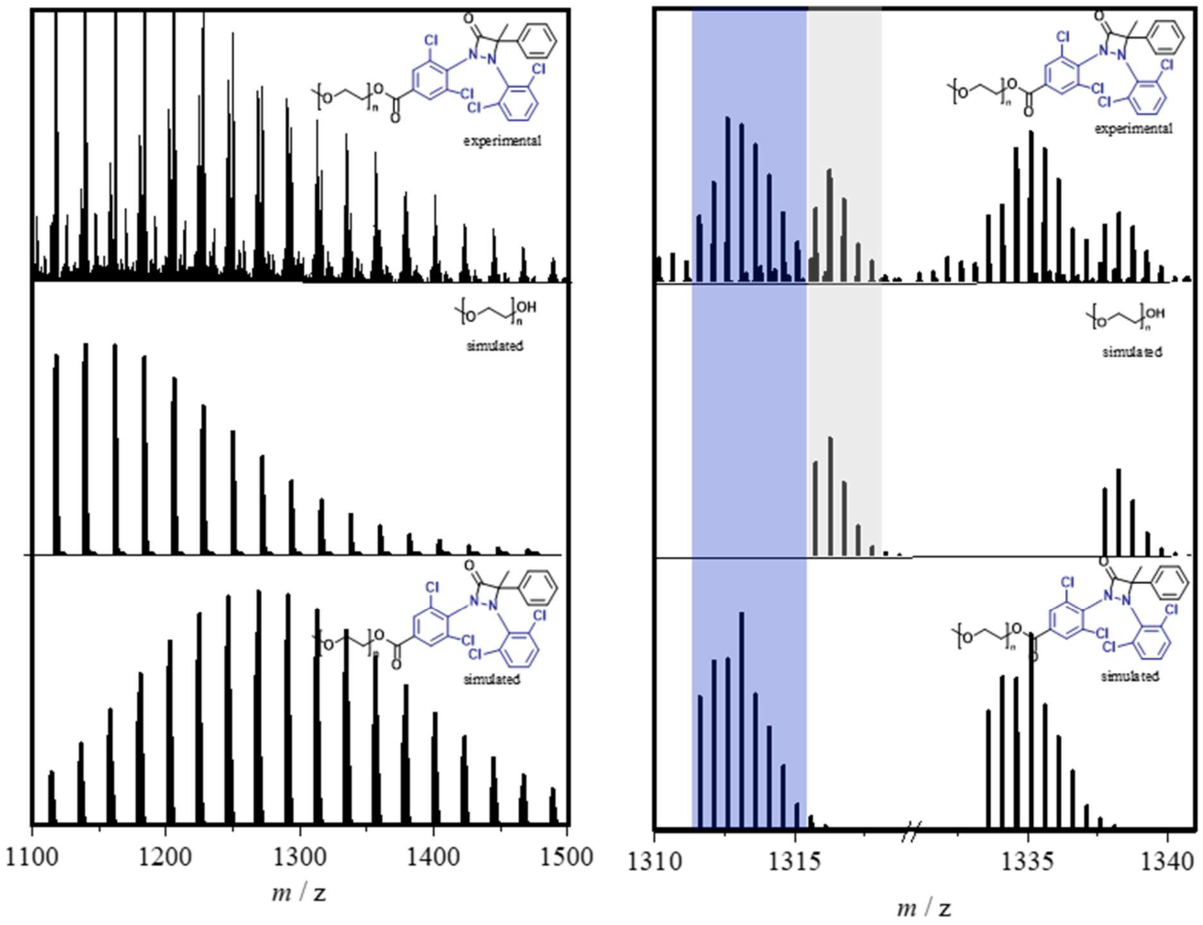

Figure 57 Comparison of the experimental SEC-MS spectra of **P3** with the simulated spectra of **P3** (blue). Additionally, the simulated spectrum of PEG-OH is depicted, matching the residual pattern (grey) in the experimental spectra of **P3**. The right zoom-in highlights a representative area and allows for precise comparison of the isotopic pattern.

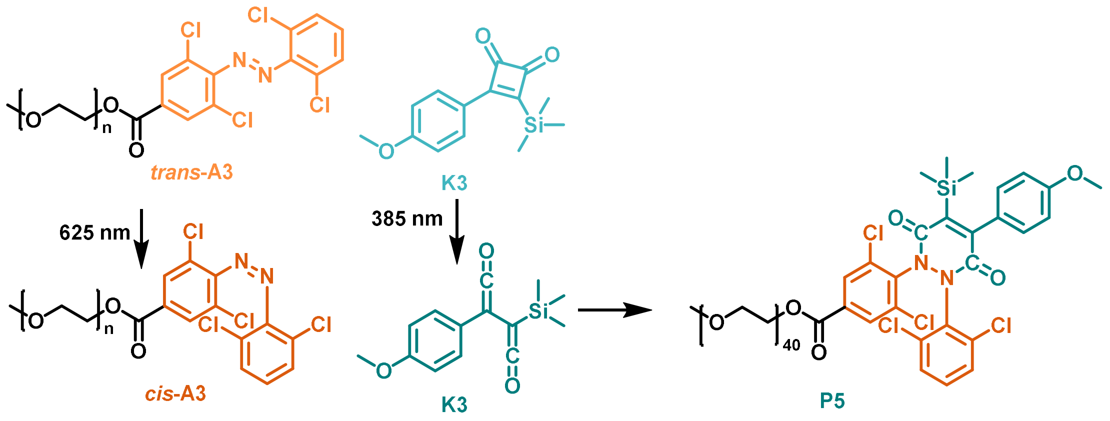

For the polymer endgroup modification, poly(ethylene glycol) azobenzene **A3** (5.0 mg, 2.2 µmol, 1.0 eq) and ketene **K3** (3.0 mg, 11.4 µmol, 5.0 eq) were dissolved in deuterated chloroform (0.2 mL) in a crimped vial. The solution was irradiated for 15 min simultaneously with 385 nm (1 A, 20 V, 2 cm distance) from one side 625 nm (2.1 A, 22 V, 2 cm distance) from the other side. For the control experiments, the same setup was used while only one wavelength turned on, respectively. Upon completion the solvent was removed under reduced pressure and the polymer was analysed by size-exclusion chromatography (SEC) and size-exclusion chromatography mass spectrometry (SEC-MS).

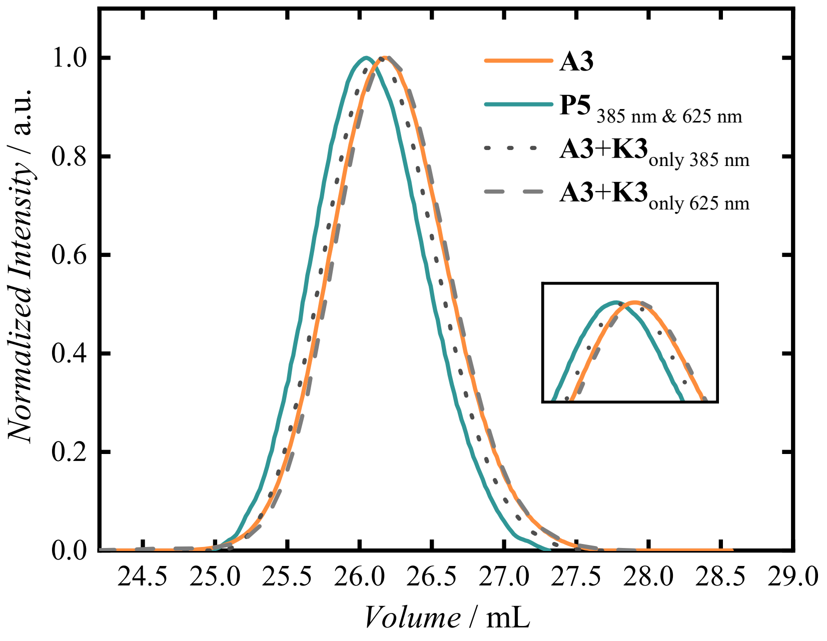

Figure 58 Size-exclusion chromatography of **P5** and after irradiation of **A3** and ketene **K3** with 385 and 625 nm. Control experiments were conducted using one wavelength, respectively.

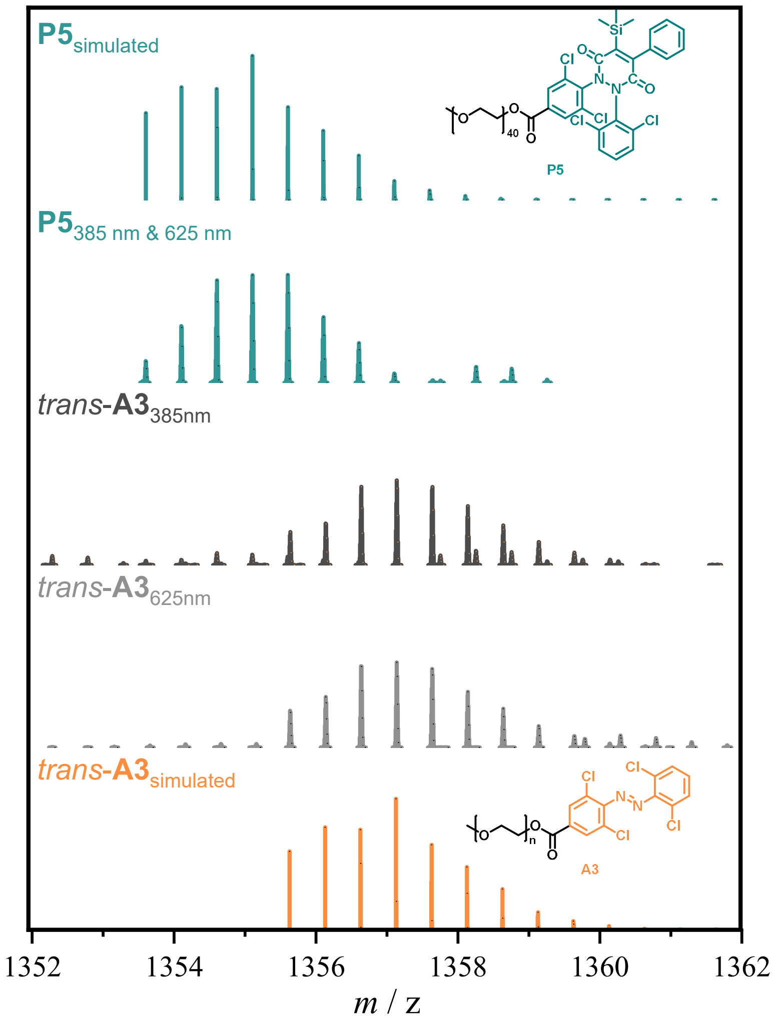

Figure 59 *top to bottom:* Comparison of the simulated spectra of **P5** with the experimental SEC-MS spectra of **P5$_{385nm\&625nm}$** (turquoise). In grey the control experiments of single LED irradiation are displayed, 385 nm or 625 nm respectively. The orange isotopic pattern represents the simulated spectra of **A3** before irradiation.

For the polymer polymer ligation, poly(ethylene glycol) azobenzene **A3** (5.0 mg, 2.2 µmol, 1.0 eq) and diethylene glycol ketene **K5** (0.8mg, 11.4 µmol, 1.5 eq) were dissolved in deuterated DCM (0.6 mL) in a NMR tube. The solution was irradiated for 30 min simultaneously with 385 nm (1 A, 20 V, 2 cm distance) from one side 625 nm (2.1 A, 22 V, 2 cm distance) from the other side. For the control experiments, the same setup was used while only one wavelength was turned on, respectively. Upon completion, the solvent was removed under reduced pressure and the polymer was analysed by size-exclusion chromatography (SEC).

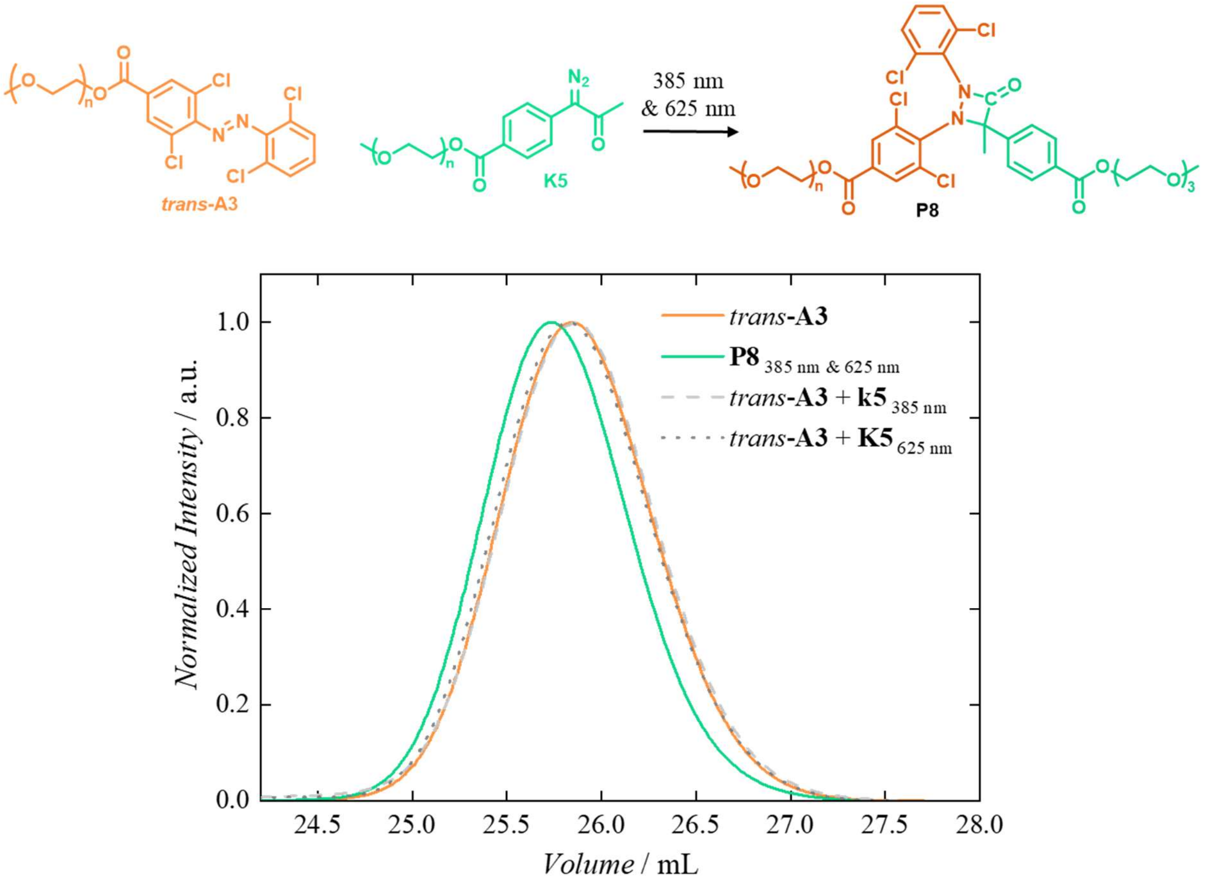

Figure 60 Size-exclusion chromatography of **P8** and after irradiation of **A3** and **K5** with 385 and 625 nm. Control experiments were conducted using one wavelength, respectively.

References

1. Konrad, D. B. *et al.* Computational Design and Synthesis of a Deeply Red-Shifted and Bistable Azobenzene. *J. Am. Chem. Soc.* **142**, 6538–6547 (2020).

2. Zhang, Z., Zhang, S., Zhang, J., Zhu, L. & Qu, D. Solvent-dependent self-assembly and morphological transition of low-molecular-weight azobenzene organogel. *Tetrahedron* **73**, 4891–4895 (2017).

3. Konrad, D. B., Frank, J. A. & Trauner, D. Synthesis of Redshifted Azobenzene Photoswitches by Late-Stage Functionalization. *Chem. - A Eur. J.* **22**, 4364–4368 (2016).

4. Ge, J. J. *et al.* Transition-Metal-Free Deacylative Cleavage of Unstrained C(sp3)-C(sp2) Bonds: Cyanide-Free Access to Aryl and Aliphatic Nitriles from Ketones and Aldehydes. *Org. Lett.* **18**, 228–231 (2016).

5. Yang, J., Ke, C., Zhang, D., Liu, X. & Feng, X. Enantioselective Synthesis of 2,2,3-Trisubstituted Indolines via Bimetallic Relay Catalysis of α-Diazoketones with Enones. *Org. Lett.* **20**, 4536–4539 (2018).

6. Xu, Y. P., Hu, R. H. & Cai, M. Z. A facile synthesis of terminal arylacetylenes via Sonogashira coupling reactions catalyzed by MCM-41-supported mercapto palladium(0) complex. *Chinese Chem. Lett.* **19**, 783–787 (2008).

7. Liu, R. & Tidwell, T. T. Ronghua Liu and Thomas T. Tidwell. **1822**, 1818–1822 (1995).

8. Loebach, J. L., Bennett, D. M. & Danheiser, R. L. (Trialkylsilyl)vinylketenes: Synthesis and application as diene components in Diels-Alder cycloadditions. *J. Org. Chem.* **63**, 8380–8389 (1998).

9. Moser, W. H., Feltes, L. A., Sun, L., Giese, M. W. & Farrell, R. W. Stereoselective [4 + 1] annulation reactions with silyl vinylketenes derived from fischer carbene complexes. *J. Org. Chem.* **71**, 6542–6546 (2006).

10. Rodriguez, K. X., Kaltwasser, N., Toni, T. A. & Ashfeld, B. L. Rearrangement of an Intermediate Cyclopropyl Ketene in a RhII-Catalyzed Formal [4 + 1]-Cycloaddition Employing Vinyl Ketenes as 1,4-Dipoles and Donor-Acceptor Metallocarbenes. *Org. Lett.* **19**, 2482–2485 (2017).

11. Fong, D. & Adronov, A. Investigation of Hybrid Conjugated/Nonconjugated Polymers for Sorting of Single-Walled Carbon Nanotubes. *Macromolecules* **50**, 8002–8009 (2017).

12. He, C., Guo, S., Huang, L. & Lei, A. Copper catalyzed arylation/C-C bond activation: An approach toward α-aryl ketones. *J. Am. Chem. Soc.* **132**, 8273–8275 (2010).
